# Correlation of phenotype with genotype and protein structure in *RYR1*-related disorders

**DOI:** 10.1007/s00415-018-9033-2

**Published:** 2018-08-28

**Authors:** Joshua J. Todd, Vatsala Sagar, Tokunbor A. Lawal, Carolyn Allen, Muslima S. Razaqyar, Monique S. Shelton, Irene C. Chrismer, Xuemin Zhang, Mary M. Cosgrove, Anna Kuo, Ruhi Vasavada, Minal S. Jain, Melissa Waite, Dinusha Rajapakse, Jessica W. Witherspoon, Graeme Wistow, Katherine G. Meilleur

**Affiliations:** 1 Neuromuscular Symptoms Unit, Tissue Injury Branch, National Institute of Nursing Research, National Institutes of Health, 10 Center Drive, Room 2A07, Bethesda, MD 20892 USA; 2 Section on Molecular Structure and Functional Genomics, National Eye Institute, National Institutes of Health, Bethesda, MD USA; 3 Mark O. Hatfield Clinical Research Center, Rehabilitation Medicine Department, National Institutes of Health, Bethesda, MD USA

**Keywords:** Genotype-phenotype, Structure-function, RyR1, Neuromuscular disease, Myopathy

## Abstract

Variants in the skeletal muscle ryanodine receptor 1 gene (*RYR1*) result in a spectrum of *RYR1*-related disorders. Presentation during infancy is typical and ranges from delayed motor milestones and proximal muscle weakness to severe respiratory impairment and ophthalmoplegia. We aimed to elucidate correlations between genotype, protein structure and clinical phenotype in this rare disease population. Genetic and clinical data from 47 affected individuals were analyzed and variants mapped to the cryo-EM RyR1 structure. Comparisons of clinical severity, motor and respiratory function and symptomatology were made according to the mode of inheritance and affected RyR1 structural domain(s). Overall, 49 *RYR1* variants were identified in 47 cases (dominant/de novo, *n* = 35; recessive, *n* = 12). Three variants were previously unreported. In recessive cases, facial weakness, neonatal hypotonia, ophthalmoplegia/paresis, ptosis, and scapular winging were more frequently observed than in dominant/de novo cases (all, *p* < 0.05). Both dominant/de novo and recessive cases exhibited core myopathy histopathology. Clinically severe cases were typically recessive or had variants localized to the RyR1 cytosolic shell domain. Motor deficits were most apparent in the MFM-32 standing and transfers dimension, [median (IQR) 85.4 (18.8)% of maximum score] and recessive cases exhibited significantly greater overall motor function impairment compared to dominant/de novo cases [79.7 (18.8)% vs. 87.5 (17.7)% of maximum score, *p* = 0.03]. Variant mapping revealed patterns of clinical severity across RyR1 domains, including a structural plane of interest within the RyR1 cytosolic shell, in which 84% of variants affected the bridging solenoid. We have corroborated genotype-phenotype correlations and identified RyR1 regions that may be especially sensitive to structural modification.

**Electronic supplementary material:**

The online version of this article (10.1007/s00415-018-9033-2) contains supplementary material, which is available to authorized users.

## Introduction

First described as a single entity in 1956 [43], congenital myopathies are now considered a spectrum of rare, slowly-progressive neuromuscular disorders with overlapping symptoms and histopathology [31]. Congenital myopathies have been attributed to pathogenic variants in over 20 genes. Of these, *RYR1*-related disorders (*RYR1*-RD) are the most frequent, identified in 90% of central core disease (CCD) patients, and with a pediatric incidence of at least 1:90,000 within the United States [3, 10, 89]. *RYR1* (19q 13.2) contains 106 exons and encodes the skeletal muscle isoform of the largest known ion channel in humans, RyR1 [89]. An autosomal dominant/de novo (AD/DN) mode of inheritance is most frequently associated with malignant hyperthermia susceptibility (MHS) whereas autosomal recessive (AR) cases often present with a more severe clinical phenotype from birth. However, malignant hyperthermia (MH) crises have also been reported, albeit less often, in AR cases and therefore all *RYR1*-RD affected individuals should be considered as potentially susceptible [1, 33, 40]. Disease manifestations include delayed motor milestones, proximal/axial muscle weakness, hypotonia, scoliosis and, in more severe cases, ophthalmoplegia and respiratory insufficiency [87]. *RYR1*-RD subtypes have classically been defined according to skeletal muscle histopathology. Examples include CCD, multi-minicore disease (MmD), centronuclear myopathy (CNM), core-rod myopathy (CRM), and congenital fiber-type disproportion (CFTD) [53]. However, these histopathological features are not unique to *RYR1*-RD, and are variable over time. In addition, there is an expanding spectrum of *RYR1*-associated clinical phenotypes, including *RYR1* rhabdomyolysis-myalgia syndrome, atypical periodic paralysis, and King-Denborough syndrome [15, 48, 88].

Forming an exceptionally large, 2.2 MDa homotetramer, RyR1 is localized to the sarcoplasmic reticulum (SR) of skeletal muscle and functions to release sarcoplasmic calcium (Ca^2+^) stores into the cytosol upon depolarization of the neuromuscular junction, enabling excitation–contraction coupling [77]. The largest RyR1 domain is the cytosolic shell (CS), also referred to as the RyR1 foot region, which constitutes the first 3613 amino acid residues and is immersed in the intracellular myoplasm [13]. The CS forms crucial inter-subunit interactions and houses the binding sites for the channel activity regulatory proteins calmodulin, S100A1 and the 12-kDa FK506-binding protein (FKBP12) [24, 57, 92]. The remaining 1423 residues constitute the channel and activation core (CAC) domain, through which SR Ca^2+^ efflux occurs and where Ca^2+^, ryanodine, and adenosine triphosphate (ATP) bind at the zinc finger-containing C-terminal region [13]. Importantly, rather than directly triggering RyR1 opening, binding of agonists such as Ca^2+^, ATP, and caffeine shift RyR1 into a primed state by decreasing the energetic resistance of specific CAC regions that are collectively termed the “activation module” [13]. In recent years, functional studies have shed light on the mechanistic consequence of specific *RYR1* variants, although these constitute < 10% of almost 700 known *RYR1* variants [28, 40]. Variants associated with *RYR1*-RD have been identified throughout the *RYR1* coding and intronic regions and can lead to chronic SR Ca^2+^ leak, decreased RyR1 protein levels, and RyR1 hyper- or hypo-sensitivity to agonists such as 4-chloro-*m*-cresol and caffeine [71, 72, 83, 95].

The last prospective genotype-phenotype assessment of *RYR1*-RD, which encompassed AD/DN and AR cases, was published over a decade ago and provided excellent insight at that time [93]. Nevertheless, numerous additional clinical phenotypes have since emerged onto the *RYR1*-RD disease spectrum, and our understanding of genotype-phenotype correlations has continued to evolve. Moreover, recent cryo-electron microscopy (cryo-EM) breakthroughs have elucidated the molecular RyR1 structure at near-atomic resolution, which has modified our understanding of established structural regions [13, 89]. Here, we use the latest cryo-EM domain/region terminology [13]. More precise localization of critical modulatory protein binding sites has also been achieved. These include sites for FKBP12 at the interface of several regions termed the bridging solenoid (Bsol), SP1a/ryanodine receptor domain 1 (SPRY1), and SP1a/ryanodine receptor domain 2 (SPRY2) regions [65, 91]. Whilst studies have revealed that AR cases are typically more clinically severe, less is known about the impact of variant location on channel function and the resulting clinical phenotype.

Using prospective data obtained from 47 *RYR1*-RD affected individuals; we sought to elucidate the complex genotype-phenotype and protein structure-phenotype relationships of this rare disease, for which there is currently no approved treatment. A detailed genotype-phenotype relationship is provided by mode of inheritance, and an assessment of clinical manifestations and severity is also made according to the affected RyR1 structural domain(s). In total, 46 variants in the *RYR1* coding region and 3 at intronic/splice sites are discussed; the former are mapped to the latest cryo-EM RyR1 structure and presented alongside published functional assay results.

## Materials and methods

### Participants

A total of 47 individuals [males, *n* = 20 (43%); adults, *n* = 31 (66%)] enrolled in a combined natural history study and double-blind, randomized, placebo-controlled trial with *N* acetylcysteine, for *RYR1*-RD (NCT02362425). The sample size in the cross-sectional analysis presented here was determined by a power calculation performed for the aforementioned clinical trial. Participants were recruited through advertisements, neuromuscular clinician referral, and patient advocacy group outreach. Study procedures were approved by a National Institutes of Health (NIH) Institutional Review Board, and participants provided informed consent or assent, in accordance with the Declaration of Helsinki, before enrollment. The study was conducted at the NIH Clinical Center, Bethesda, MD, USA, between March 2015 and November 2017 and consisted of a 6-month natural history assessment and 6-month intervention. For the cross-sectional analysis presented here, data were obtained from participants at baseline. Inclusion and exclusion criteria are detailed at: NCT02362425.

### *RYR1* sequencing and variant screening

Diagnostic genetic testing reports were obtained from individuals’ medical records. Genetic testing was conducted at laboratories certified to the Clinical Laboratory Improvement Amendments (CLIA) standards, or non-U.S equivalent. Alamut Visual (version 2.9.0, Interactive Biosoftware, Rouen, France), was used to confirm *RYR1* variants specified in genetic testing reports, generate orthologue alignments, and identify previously reported variants. For missense substitutions, differences in physico-chemical properties between wild-type and mutant amino acids were also estimated using Alamut Visual [19, 67, 74]. Three-generation family histories and parental genetic testing reports, when available, were obtained from participants to confirm the mode of inheritance. When this was not possible, a plausible mode of inheritance was established through careful evaluation of clinical manifestations characteristic of AR cases [2].

### Physical examination and clinical severity grading

A single Nurse Practitioner administered all physical examinations for study participants. This included assessment of the following systems: head, ears, eyes, nose and throat, neurologic, respiratory, cardiovascular, gastrointestinal, genitourinary, endocrine, hematologic, immune, dermatologic, psychiatric, and musculoskeletal health. Distal and proximal weakness was ascertained by manual muscle testing and were defined as two or more ≤ 4 grade responses. Heat and exercise tolerance were determined using both the participant’s medical record and self-reported medical history the time of study enrolment. Clinical severity was determined using an *RYR1*-RD 8-point scale focused on ambulatory and respiratory function [2].

### Skeletal muscle histopathology

Skeletal muscle histopathology reports were obtained from participants’ medical records. Reports were available for 26/47 participants. Each panel typically included histology: NADH tetrazolium reductase (NADH-TR), hematoxylin and eosin (HE), Gömöri Trichrome (GO), periodic acid-Schiff (PAS), Oil-Red O (ORO); histo-enzymology staining: cytochrome oxidase (COX), succinate dehydrogenase (SDH), ATPase; and immunohistochemistry: myosin isoform (slow and fast heavy chain).

### Assessment of respiratory function

Pulmonary function tests (PFTs) were conducted by a physical therapist in accordance with American Thoracic Society (ATS) guidelines [35]. PFTs included forced vital capacity (FVC), forced expiratory volume at 1 s (FEV_1_), FVC to FEV_1_ ratio, and slow vital capacity (SVC). Percent predicted values for PFTs were calculated using BreezeSuite software (CPFS/D USB spirometer, MGC Diagnostics, Saint Paul, MN, USA). Thresholds of < 80 and < 60% predicted FVC were used to define respiratory insufficiency and moderate respiratory insufficiency, respectively [25, 82]. Participants on BiPAP or CoughAssist were also categorized as having impaired respiratory function.

### Motor function measure (MFM-32) assessment

Motor function was evaluated using MFM-32 which has been developed and validated for use in the neuromuscular disease population, including *RYR1*-RD [80, 81]. This was completed for each participant by physical therapists. MFM-32 consists of three dimensions that account for posture and whole-body movements related to standing and transfers (dimension 1), axial and proximal motor function (dimension 2), and distal motor function (dimension 3). Data were expressed as a percentage of the maximum possible score for each dimension as well as an overall total score.

### Variant mapping

Variant analysis and graphical representation were performed with Pymol software (version 2.0.4; Schrödinger, LLC, NY) using PDB (Protein Data Bank; [6]) structure PDB: 5TAX open state. All *RYR1* coding-region variants identified in this cohort (*n* = 46) were mapped to the RyR1 monomer based on domain location, except stop-gain (premature termination), synonymous substitution, and frame-shift variants (*n* = 8), and those affecting unassigned residues (*n* = 2). Variants were further mapped based on clinical severity using the abovementioned scale. Variants associated with clinically severe phenotypes were mapped in red. Variants associated with mild clinical severity (severity scores below 5) were subdivided into three categories: orange (severity score of 3–4), green (severity score of 1–2), and white (severity score of 0). When multiple cases were associated with a specific variant, an average clinical severity score was calculated, to the nearest whole number.

### Statistics

All statistical tests were conducted using the Statistical Package for the Social Sciences version 24 (SPSS; IBM, Armonk, NY, USA). For genotype-phenotype comparisons, participants were grouped based on mode of inheritance; AD/DN or AR. For structure-phenotype comparisons, cryo-EM-defined residue spans for RyR1 structural domains [13], were used to group participants based on whether *RYR1* variant(s) were located in the (a) only the RyR1 CS domain, (b) only the RyR1 CAC domain, or (c) both domains. Descriptive statistics were generated for each group and data distribution was assessed using the Shapiro–Wilk test. MFM-32 and age at diagnosis data were skewed, therefore Mann–Whitney *U* test or Kruskal–Wallis with Dunn’s post-hoc test were used to identify statistically significant differences between groups. Data for PFTs followed a Gaussian distribution, therefore differences between groups were assessed by ANOVA with Bonferroni post-hoc test or independent *t* test. Fisher’s Exact test was used to compare the proportion of clinically severe cases (severity score ≥ 5) by mode of inheritance, by affected RyR1 domain(s), and the proportion of cases that exhibited moderate respiratory insufficiency, by mode of inheritance and affected RyR1 domain(s).

## Results

In this cohort, 49 variants were identified, with 46 located in the *RYR1* coding region and three at intronic/splice sites (Table 1). Three variants (p.Asn4575Thr, p.Met4840Arg, and p.Met4875Val) were novel (i.e., not reported in ExAC/gnomAD, ESP, HGVD, ClinVar, 1000 Genomes, or HGMD databases and not published to date). An AD/DN mode of inheritance was most frequent (35/47 cases), and all AR cases were compound heterozygous. In this cohort, variants affected the following RyR1 domain(s): only the CS *n* = 12 cases; only the CAC *n* = 29; both domains *n* = 6 cases. Summary demographics are provided in Table 2. For participants born before the advent of massively parallel (next generation) sequencing in 2004 (*n* = 35) [79], the median (IQR) age of *RYR1*-RD diagnosis was 36.0 (23.4) years compared to 4.5 (3.8) years in those born after 2004, *p* < 0.001. Structural and functional data for each variant are detailed in Table 3. There was no difference in clinical severity scores between males (*n* = 20) versus females (*n* = 27), (average clinical severity score = 3 for both groups, *p* = 0.139).

**Table 1:** Genetic details of *RYR1*-RD affected individuals

Case:Family	Exon/intron	Nucleotide change	Amino acid change	Mode of inheritance	Variant classification^a^	Reported in
Participants with variant(s) affecting only the RyR1 cytosolic shell						
1:1	E 46	c.7354C > T	p.Arg2452Trp	Dominant	Pathogenic	[61]
2:1	E 46	c.7354C > T	p.Arg2452Trp	Dominant		
3:2	E 10	c.838C > T	p.Arg280*	Recessive	VUS	[77]
	E 66	c.9716T > A	p.Met3239Lys		VUS	[77]
4:3	E 41	c.6697T > C	p.Cys2233Arg	Dominant	VUS	[77]
5:4	E 41	c.6721C > T	p.Arg2241*	Recessive	Pathogenic	[27]
	E 4	c.325C > T	p.Arg109Trp		Likely pathogenic	[95]
	E 18	c.2122G > A	p.Asp708Asn		VUS	[86]
	E 14	c.1453A > G	p.Met485Val		VUS	[95]
6:5	E 39	c.6488G > A	p.Arg2163His	Dominant	Pathogenic	[45]
7:6	E 15	c.1589G > A	p.Arg530His	Recessive	VUS	[96]
	E 24	c.3127C > T	p.Arg1043Cys		VUS	[96]
	E 43	c.7007G > A	p.Arg2336His		VUS	[7]
8:7	E 24	c.2923C > T	p.Arg975Trp	Dominant	VUS	[9]
9:7	E 24	c.2923C > T	p.Arg975Trp	Dominant		
10:8	E 44	c.7166_7176del11	p.Asp2389Glyfs*16	Clinically recessive^b^	Likely pathogenic	[77]
	I 58	c.8933-1G > A	(intronic)		Likely pathogenic	[76]
11:9	E 31	c.4485_4500del16	p.Trp1495*	Recessive	Pathogenic	[8]
	E 44	c.7060_7062delGTG	p.Val2354del		Likely pathogenic	[8]
12:10	E 40	c.6617C > T	p.Thr2206Met	Recessive	Pathogenic	[62]
	I 59	c.9001-2A > G	(intronic)		VUS	[70]
Participants with variant(s) affecting only the RyR1 channel and activation core						
13:11	E 94	c.13724A > C	p.Asn4575Thr	Dominant	VUS	This manuscript
14:12	E 102	c.14763C > G	p.Phe4921Leu	Dominant	VUS	[77]
15:13	E 102	c.14693T > C	p.Ile4898Thr	Dominant	Pathogenic	[23]
16:13	E 102	c.14693T > C	p.Ile4898Thr	Dominant		
17:14	E 103	c.14818G > A	p.Ala4940Thr	Dominant	Pathogenic	[59]
18:14	E 103	c.14818G > A	p.Ala4940Thr	Dominant		
19:15	E 103	c.14818G > A	p.Ala4940Thr	Dominant		
20:16	E 100	c.14458G > A	p.Gly4820Arg	Dominant	VUS	[36]
21:16	E 100	c.14458G > A	p.Gly4820Arg	Dominant		
22:16	E 100	c.14458G > A	p.Gly4820Arg	Dominant		
23:17	E 101	c.14582G > A	p.Arg4861His	Dominant	Pathogenic	[70]
24:17	E 101	c.14582G > A	p.Arg4861His	Dominant		
25:18	E 101	c.14582G > A	p.Arg4861His	Dominant		
26:19	E 101	c.14582G > A	p.Arg4861His	Dominant		
27:20	E 101	c.14582G > A	p.Arg4861His	Dominant		
28:21	E102	c.14678 G > A	p.Arg4893Gln	Dominant	Pathogenic	[12]
29:21	E102	c.14678 G > A	p.Arg4893Gln	Dominant		
30:22	E 102	c.14681C > A	p.Ala4894Asp	Dominant	VUS	[77]
31:22	E 102	c.14681C > A	p.Ala4894Asp	Dominant		
32:23	E 101	c.14582G > A	p.Arg4861His	Clinically dominant^b^	Pathogenic	[69]
	E 91	c.13331_13351dup	p.Gly4444-		VUS	gnomAD# 19:39056300
33:24	E 103	c.14807T > G	p.Leu4936Arg	Dominant	VUS	[2]
34:25	E 98	c.14210G > A	p.Arg4737Gln	Clinically recessive^b^	Pathogenic	[17]
	E 88	c.12063_12064dupCA	p.Met4022Thrfs*4		VUS	LOVD# 0030253
	I 41	c.6797-9C > T	(intronic)		Likely benign	dbSNP# 191934693
35:26	E 88	c.12083C > T	p.Ser4028Leu	Dominant	VUS	[11]
36:27	E 100	c.14422_14423delTTinsAA	p.Phe4808Asn	Dominant	Likely pathogenic	[12]
37:28	E 101	c.14558C > T	p.Thr4853Ile	Dominant	Pathogenic	[21]
38:29	E 102	c.14731G > A	p.Glu4911Lys	Dominant	Pathogenic	[7]
39:30	E 92	c.13513G > C	p.Asp4505His	Dominant	VUS	[11]
40:30	E 92	c.13513G > C	p.Asp4505His	Dominant		
41:30	E 92	c.13513G > C	p.Asp4505His	Dominant		
Participants with variant(s) affecting both the RyR1 cytosolic shell and channel and activation core						
42:31	E 43	c.7025A > G	p.Asn2342Ser	Recessive	VUS	[46]
	E 101	c.14519T > G	p.Met4840Arg		VUS	This manuscript
43:29	E 102	c.14731G > A	p.Glu4911Lys	Recessive	Pathogenic	[33]
	E 33	c.4711A > G	p.Ile1571Val		VUS	[70]
	E 67	c.10097G > A	p.Arg3366His		VUS	[73]
	E 86	c.11798A > G	p.Tyr3933Cys		VUS	[7]
44:32	E 41	c.6721C > T	p.Arg2241*	Clinically recessive^b^	Pathogenic	[7]
	E 96	c.14126C > T	p.Thr4709Met		Pathogenic	[76]
45:15	E 41	c.6671G > A	p.Arg2224His	Recessive	VUS	dbSNP# 537994744
	E 103	c.14818G > A	p.Ala4940Thr		Pathogenic	[59]
46:33	E 45	c.7300G > A	p.Gly2434Arg	Clinically recessive^b^	Pathogenic	[14]
	E 101	c.14623A > G	p.Met4875Val		VUS	This manuscript
47:34	E26	c.3495C > T	p.Gly1165Gly	Recessive	VUS	dbSNP# 772616442
	E33	c.4817G > A	p.Arg1606His		VUS	dbSNP# 368399715
	E90	c.12499G > T	p.Glu4167*		Pathogenic	dbSNP# 772494345

*E* exon number, *I* intron number, *LOVD* Leiden Open (source) Variation Database, *dbSNP* single nucleotide polymorphism database, *gnomAD* The Genome Aggregation Database, *VUS* variant of uncertain significance

^a^Determined by genetic testing reports and validation using Alamut Visual

^b^Such cases did not have parental genetic testing, therefore, a plausible mode of inheritance was established through careful evaluation of clinical manifestations

**Table 2:** Summary demographics of the *RYR1*-RD affected individuals

Measure	Total cohort	Mode of inheritance		Affected RyR1 domain(s)		
	(*n* = 47)	AD/DN (*n* = 35)	AR (*n* = 12)	CS (*n* = 12)	CAC (*n* = 29)	Both domains (*n* = 6)
Age at enrolment, years	28.6 ± 17.3^a^	31.7 ± 17.2	20.8 ± 15.2	29.1 ± 17.9	31.3 ± 17.3	15.2 ± 10.3
Age at *RYR1*-RD diagnosis, years	22.3 ± 10.1	29.1 ± 17.9	18.2 ± 15.5	26.6 ± 18.1	29.5 ± 17.7	10.7 ± 9.5
Sex, ♂/♀	20:27^b^	15:20	5:7	5:7	13:16	2:4
Pediatric/adult	16:31	10:25	6:6	4:8	8:21	4:2
Height, cm	154.1 ± 20.0	156.4 ± 19.8	148.7 ± 20.0	154.4 ± 20.2	155.4 ± 19.1	147.6 ± 25.9
Weight, kg	57.0 ± 27.9	62.4 ± 29.1	45.2 ± 23.1	62.3 ± 32.7	59.0 ± 26.1	36.8 ± 20.0
BMI, kg/m^2^	22.7 ± 8.3	24.2 ± 8.8	19.2 ± 6.0	24.6 ± 9.8	23.3 ± 7.7	15.9 ± 3.6

*AD/DN* autosomal dominant/de novo, *AR* autosomal recessive, *CS* only the RyR1 CS affected, *CAC* only the RyR1 CAC affected

^a^Data are expressed as mean ± SD

^b^Data are expressed as frequency

**Table 3:** Affected RyR1 region(s), physico-chemical changes for missense substitutions and functional studies

Case:Family	*RYR1* variant		Affected RyR1 region	Affected RyR1 functional site	Change in amino acid composition (AU)	Change in amino acid polarity (AU)	Change in amino acid molecular volume (AU)	Grantham distance (AU)	Functional studies	References
Participants with variant(s) affecting only the RyR1 cytosolic shell										
1:1	p.Arg2452Trp	Bsol		MH/CCD hotspot 2	0.52	5.1	46	101	↓ 4-C*m*C threshold; +IVCT	[5, 60, 71, 90]
2:1	p.Arg2452Trp			Bsol-NTD interface						
3:2	p.Arg280* ^a^	NTD-B		MH/CCD hotspot 1	n/a	n/a	n/a	n/a	None	[68]
	p.Met3239Lys	Bsol		–	0.33	5.6	14	95	None	
4:3	p.Cys2233Arg	Bsol		MH/CCD hotspot 2	2.1	5.0	69	180	None	[49]
5:4	p.Arg2241* ^a^	Bsol		MH/CCD hotspot 2	n/a	n/a	n/a	n/a	DHPR/RyR1 misalignment, ↓ RyR1	[26, 49, 91, 94, 95]
	p.Arg109Trp	NTD-A		MH/CCD hotspot 1	0.52	5.1	46	101	↓ RyR1	
				NTD-A-Nsol interface						
	p.Asp708Asn	SPRY1		Protein–protein interaction motif; crucial FKBP interaction site	0.05	1.4	2	23	DHPR/RyR1 misalignment, ↓ RyR1	
				MH/CCD hotspot 1						
	p.Met485Val	Nsol		Nsol-NTD interface	0.0	0.2	21	21	↓ RyR1 (in conjunction with p.Arg109Trp)	
6:5	p.Arg2163His	Bsol		MH/CCD hotspot 2, close to FKBP12 binding site at the Bsol (BrA), SPRY1, and SPRY2 junctional interface	0.07	0.1	28	29	+IVCT	[45, 49, 65]
7:6	p.Arg530His	Nsol		MH/CCD hotspot 1	0.07	0.1	28	29	↑ acidification rate post-4-C*m*C	[39, 49, 91, 96]
	p.Arg1043Cys	RY1&2		RY1&2-Csol interface					+IVCT	
				Inter-subunit interaction site	2.1	5.0	69	180		
				Bsol-SPRY3 interface						
	p.Arg2336His	Bsol		MH/CCD hotspot 2	0.07	0.1	28	29	↓ 4-C*m*C threshold versus WT	
8:7	p.Arg975Trp	RY1&2		Inter-RyR1 interaction site	0.52	5.1	46	101	None	[91]
9:7	p.Arg975Trp			RY1&2-SPRY3 interface						
10:8	p.Asp2389Glyfs*16^a^	Bsol		MH/CCD hotspot 2	n/a	n/a	n/a	n/a	None	[49]
11:9	p.Trp1495*^a^	SPRY3		Direct contact with NTD; involved in	n/a	n/a	n/a	n/a	None	[4, 49]
				RyR1-Cav1.1 coupling						
	p.Val2354del	Bsol		MH/CCD hotspot 2	n/a	n/a	n/a	n/a	None	
				Bsol-NTD interface						
12:10	p.Thr2206Met	Bsol		MH/CCD hotspot 2	0.71	2.9	44	81	↓ 4-C*m*C threshold versus WT + IVCT	[49, 62, 83]
Participants with variant(s) affecting only the RyR1 channel and activation core										
13:11	p.Asn4575Thr		pVSD	MH/CCD hotspot 3, linked to S2S3 and critical to RyR1 opening	0.62	3	5	65	None	[84, 89]
				pVSD-Pore interface						
14:12	p.Phe4921Leu		Pore	MH/CCD hotspot 3, residue binds ryanodine	0.0	0.3	21	22	None	[52, 89]
15:13	p.Ile4898Thr		Pore	MH/CCD hotspot 3, luminal triadin binding, retention of RyR-CSQ proximity and ability for rapid Ca^2+^ release, selectivity filter	0.71	3.4	50	89	↓ caffeine-induced Ca^2+^ release, ↑ 4-C*m*C threshold, ↓ Ca^2+^ stores compared to WT and other *RYR1* variants, ↓/↑ resting cytosolic Ca^2+^	[37, 38, 42, 66, 75, 89]
16:13	p.Ile4898Thr									
17:14	p.Ala4940Thr		Pore (S6c)	MH/CCD hotspot 3, ATP binding site, close to C-terminus	0.71	0.5	30	58	+IVCT	[13, 63, 89]
18:14	p.Ala4940Thr									
19:15	p.Ala4940Thr									
20:16	p.Gly4820Arg		Pore	MH/CCD hotspot 3					+IVCT	[36, 89]
21:16	p.Gly4820Arg			Pore-pVSD interface	0.09	1.5	121	125		
22:16	p.Gly4820Arg									
23:17	p.Arg4861His		Pore	MH/CCD hotspot 3, luminal triadin binding, retention of RyR-CSQ proximity and ability for rapid Ca^2+^ release	0.07	0.1	28	29	↑ Ca^2+^ release pre-treatment, ↓ thapsigargin-induced Ca^2+^ release, ↓ Ca^2+^ release in response to 4-C*m*C, ↓ 4-C*m*C threshold than WT, + IVCT	[37, 38, 55, 68, 75]
24:17	p.Arg4861His									
25:18	p.Arg4861His									
26:19	p.Arg4861His									
27:20	p.Arg4861His			Pore-pVSD interface						
28:21	p.Arg4893Gln		Pore	MH/CCD hotspot 3, luminal triadin binding, adjacent to ryanodine-binding residue, retention of RyR-CSQ proximity and ability for rapid Ca^2+^ release	0.24	0.0	39	43	EC-uncoupling, ↑ 4-C*m*C threshold than WT	[34, 37, 38, 52, 89]
29:21	p.Arg4893Gln									
30:22	p.Ala4894Asp		Pore	MH/CCD hotspot 3, luminal triadin binding, close to ryanodine-binding residue, retention of RyR-CSQ proximity and ability for rapid Ca^2+^ release, critical for Ca^2+^ selectivity	1.38	4.9	23	126	None	[22, 37, 38, 52, 89]
31:22	p.Ala4894Asp									
32:23	p.Arg4861His		Pore	MH/CCD hotspot 3, luminal triadin and ryanodine binding, retention of RyR-CSQ proximity and ability for rapid Ca^2+^ release	0.07	0.1	28	29	↑ Ca^2+^ release pre-treatment, ↓ thapsigargin-induced Ca^2+^ release, ↓ Ca^2+^ release in response to 4-C*m*C, ↓ 4-C*m*C threshold than WT, + IVCT	[37, 38, 55, 68, 75]
	p.Gly4444-Gly4450dup		–	MH/CCD hotspot 3, S100A1 binding site, influences CaM activity	n/a	n/a	n/a	n/a	None	[57, 78, 89]
33:24	p.Leu4936Arg		Pore	MH/CCD hotspot 3; directly adjacent to the critical gating residue p.Ile4937	0.65	5.6	13	102	None	[85, 89]
34:25	p.Arg4737Gln		pVSD	MH/CCD hotspot 3	0.24	0.0	39	43	None	[89]
				pVSD-Pore interface						
	p.Met4022Thrfs*4^a^		Csol	Linked to S2S3 and critical to RyR1 opening, close to putative Ca^2+^ binding site	n/a	n/a	n/a	n/a	None	[13, 84]
35:26	p.Ser4028Leu		Csol	Close to putative Ca^2+^ binding site	1.42	4.3	79	145	None	[13, 51]
				Csol-CTD interface						
36:27	p.Phe4808Asn		pVSD	MH/CCD hotspot 3, linked to S2S3 and critical to RyR1 opening	1.33	6.4	76	158	None	[84, 89]
				pVSD-TMx interface						
37:28	p.Thr4853Ile		Pore	MH/CCD hotspot 3	0.71	3.4	50	89	+IVCT	[21, 89]
				Pore-pVSD interface						
38:29	p.Glu4911Lys		Pore	MH/CCD hotspot 3, luminal triadin binding	0.59	1.0	36	56	None	[38, 89]
				Pore-TMx interface						
39:30	p.Asp4505His		–	MH/CCD hotspot 3, S100A1 binding site, influences CaM activity	0.8	2.6	42	81	↑ caffeine-induced Ca^2+^ release, + IVCT	[20, 57, 78, 89]
40:30	p.Asp4505His									
41:30	p.Asp4505His									
Participants with variant(s) affecting both the RyR1 cytosolic shell and channel and activation core										
42:31	p.Asn2342Ser		CS	MH/CCD hotspot 2	0.09	2.4	24	46	↑ acidification rate post-4-C*m*C treatment	[13, 49, 96]
			(Bsol)	Inter-subunit contact with NTD-A						
	p.Met4840Arg		CAC (Pore)	MH/CCD hotspot 3, channel stabilizing inter-domain interaction	0.65	4.8	19	91	None	[26, 89]
43:29	p.Glu4911Lys		CAC (Pore)	MH/CCD hotspot 3, luminal triadin binding	0.59	1.0	36	56	None	[38, 89]
	p.Ile1571Val		CS (SPRY3)	Currently unassigned residue	0.0	0.7	27	29	None	[13]
	p.Arg3366His		CS (Bsol)	Currently unassigned residue	0.07	0.1	28	29	None	[13]
	p.Tyr3933Cys		CAC (Csol)	Close to putative Ca^2+^ binding site	2.55	0.7	81	194	None	[13]
				Csol-SPRY3 interface						
44:32	p.Arg2241*^a^		CS (Bsol)	MH/CCD hotspot 2	n/a	n/a	n/a	n/a	DHPR/RyR1 misalignment, ↓ RyR1	[49, 94]
	p.Thr4709Met		CAC [pVSD (S2S3)]	MH/CCD hotspot 3, linked to S2S3 and critical to RyR1 opening	0.71	2.9	44	81	↓ RyR1	[84, 89, 93]
45:15	p.Arg2224His		CS (Bsol)	MH/CCD hotspot 2	0.07	0.1	28	29	None	[49]
	p.Ala4940Thr		CAC [Pore (S6c)]	MH/CCD hotspot 3, next to conserved hinge glycine associated with RyR1 opening. Critical for Ca^2+^ flow	0.71	0.5	30	58	+IVCT	[50, 63, 89]
46:33	p.Gly2434Arg		CS (Bsol)	MH/CCD hotspot 2, NTD-Bsol contact (DP4 peptide)	0.09	1.5	121	125	+IVCT ↑, ryanodine binding and ↑ sensitivity to caffeine and 4C*m*C	[13, 18, 49, 58]
	p.Met4875Val		CAC (Pore)	MH/CCD hotspot 3, luminal triadin binding, retention of RyR-CSQ proximity and ability for rapid Ca^2+^ release	0.0	0.2	21	21	None	[37, 38, 89]
				Pore-TMx interface						
47:34	p.Gly1165Gly		CS (SPRY2)	Residue close to RyR1-Cav1.1 interaction site	n/a	n/a	n/a	n/a	None	[64]
	p.Arg1606His		CS (SPRY3)	Probable RyR1-Cav1.1 interaction	0.07	0.1	28	29	None	[64]
				SPRY3-RY1&2 interface						
	p.Glu4167*^a^		CAC (Csol)	MH/CCD hotspot 3	n/a	n/a	n/a	n/a	None	[89]

*CS* CS, *CSQ* calsequestrin, *CAC* CAC, *MH* malignant hyperthermia, *CCD* central core disease, *Bsol* bridging solenoid, *NTD-B* N-terminal domain B, *NTD-A* N-terminal domain A, *SPRY1* SP1a/ryanodine receptor domain 1, *Nsol* N-terminal solenoid, *RY1&2* RYR repeats 1 and 2, *SPRY3* SP1a/ryanodine receptor domain 3, *Pore* channel pore domain, *S6c* cytoplasmic extension of S6, *pVSD* pseudo voltage sensor domain, *S2S3* helical-bundle domain between S2 and S3, *IVCT* in vitro caffeine-halothane contracture test, *DHPR* dihydropyridine receptor, *4-CmC* 4-chloro-*m*-cresol, *WT* wild-type, *RyR1* ryanodine receptor isoform 1, *Ca*^*2+*^ calcium, *EC* excitation–contraction

^a^Denoted variants may be associated with decreased RyR1 expression via nonsense-mediated mRNA decay. As such, these variants likely affect overall RyR1 expression, rather than impacting only the domain within which the variant was identified

### Genotype-phenotype correlation and histopathology

**Fig. 1:**
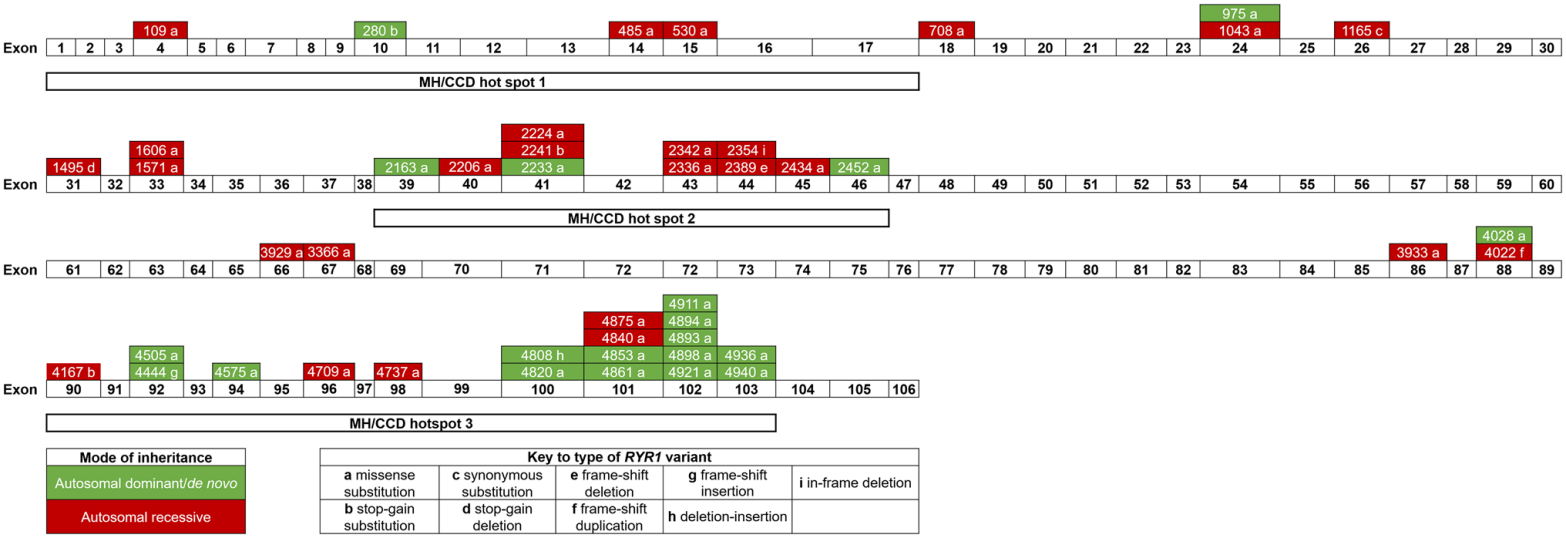
Distribution of variants across the *RYR1* coding region including MH/CCD hot spots. Numbers within green and red boxes correspond to the affected amino acid residue

*RYR1* coding region variants predominantly consisted of missense substitutions (36/46); 89.1% of which affected highly evolutionarily conserved positions (Figs. S1 and S2). Other variant types included stop-gain substitution (*n* = 3), synonymous substitution (*n* = 1), deletion leading to stop-gain (*n* = 1), frame-shift deletion/duplication/insertion (*n* = 3), deletion-insertion (*n* = 1), and in-frame deletion (*n* = 1). Three intronic substitutions were identified, two of which were canonical splice site variants (c.8933-1 G > A and c.9001-2A > G). In 94% of the cohort, *RYR1* variant(s) were identified within one or more of the three established MH/CCD hot spot regions [89]. Variant distribution across the *RYR1* coding region, including MH/CCD hot spot regions, is depicted in Fig. 1. Multiple *RYR1* variants were identified in 30% of the cohort with 13% of these participants possessing variants that affected both RyR1 domains. There was no difference in the proportion of clinically severe cases (severity score ≥ 5), by mode of inheritance (AD/AR 11% versus AR 25%, *p* = 0.35), Fig. S3A.

Overall, 73% of participants with histopathology reports (*n* = 26) had evidence of cores, Fig. S4. AD/DN cases with biopsy results (*n* = 16) were associated with CCD/MmD pathology 88% of the time with the remaining 12% exhibiting either no pathology or inconclusive biopsy results. Of note, the single case (case 39) for which no histopathology was evident on biopsy, exhibited a recurrent rhabdomyolysis-myalgia clinical phenotype. In AR cases with histopathology reports available (*n* = 10), biopsy findings were more diverse, however, CCD/MmD pathology was still most frequently observed (40% of cases), followed by congenital fiber-type disproportion (30% of cases).

**Fig. 2:**
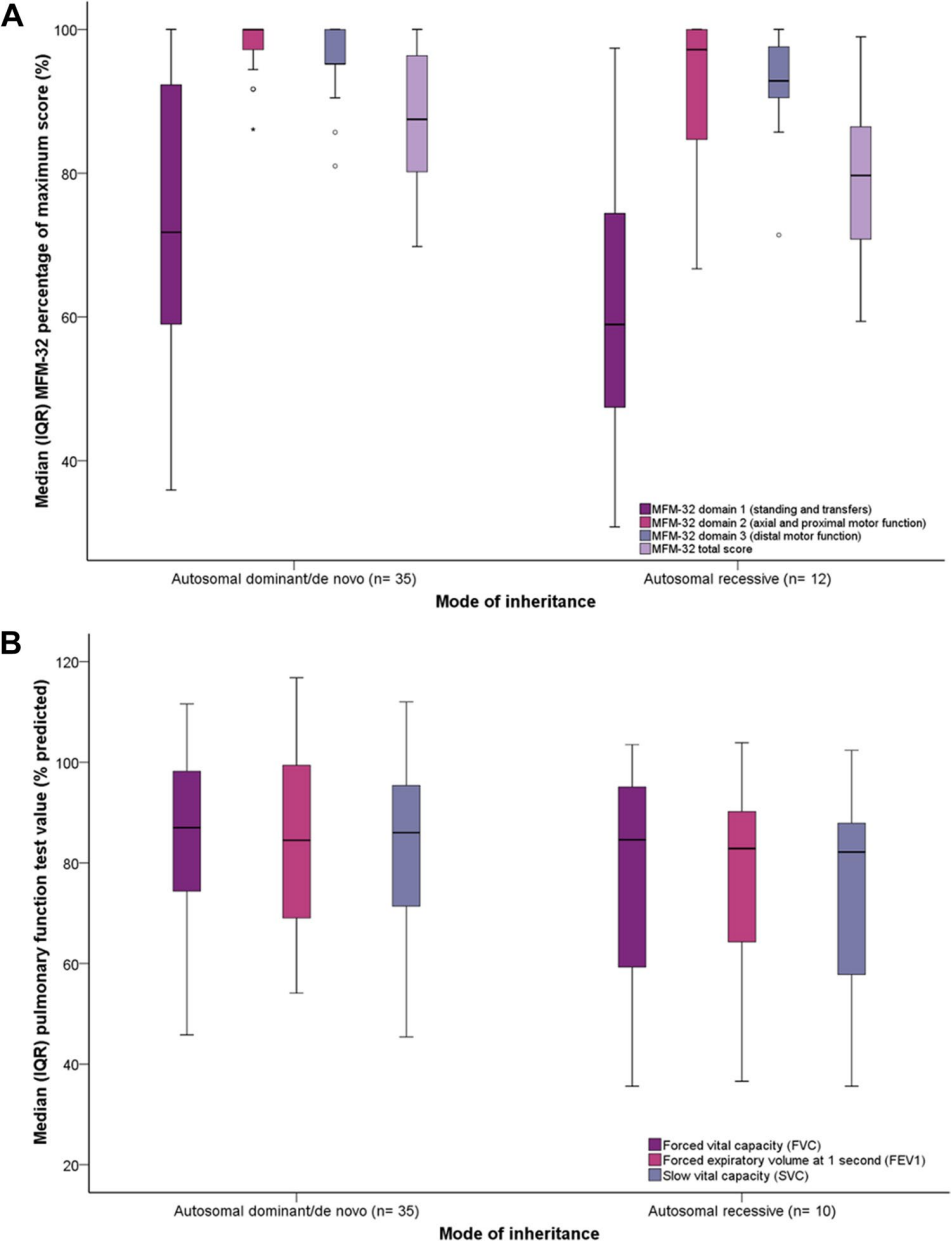
Median (IQR) MFM-32 scores (**a**) and pulmonary function test values (**b**) by mode of inheritance. **a** Except for standing and transfers, participants with an AR mode of inheritance achieved a significantly lower median (IQR) percentage of maximum MFM-32 score across all other MFM-32 domains, when compared to AD/DN cases [standing and transfers, 71.8 (33.3)% vs. 59.0 (27.6)% *p* = 0.078; axial and proximal motor function, 100.0 (19.1)% vs. 97.2 (16.0)% *p* = 0.017; distal motor function, 95.2 (4.8)% vs. 92.9 (8.3)% *p* = 0.046; total score, 87.5 (17.7)% vs. 79.7 (18.8)% *p* = 0.037]. There was no difference in pulmonary function parameters when compared by mode of inheritance (all, *p* > 0.05), (^∘^ and * denote outliers)

The overall median (IQR) MFM-32 result for this cohort (% maximum score) was as follows: standing and transfers 66.7 (35.5)%; axial and proximal motor function 100.0 (5.6)%; distal motor function 95.2 (9.5)%; total score 85.4 (18.8)%. With the exception of standing and transfers, AR cases achieved significantly lower MFM-32 score across all other MFM-32 dimensions, compared to AD/DN cases [standing and transfers, 59.0 (27.6)% vs. 71.8 (33.3)% *p* = 0.078; axial and proximal motor function, 97.2 (16.0)% vs. 100.0 (19.1)% *p* = 0.017; distal motor function, 92.9 (8.3)% vs. 95.2 (4.8)% *p* = 0.046; total score, 79.7 (18.8)% vs. 87.5 (17.7)% *p* = 0.037], Fig. 2a.

Two AR cases were unable to perform PFTs owing to tracheostomy and inability to meet all PFT standardization criteria (cases 10 and 12, respectively). Overall, 38% of the cohort exhibited respiratory insufficiency (FVC < 80% predicted) with 13% demonstrating moderate respiratory insufficiency (FVC < 60%). There was no difference in PFT results according to the mode of inheritance (all, *p* > 0.05), Fig. 2b and Fig. S5a, b.

**Fig. 3:**
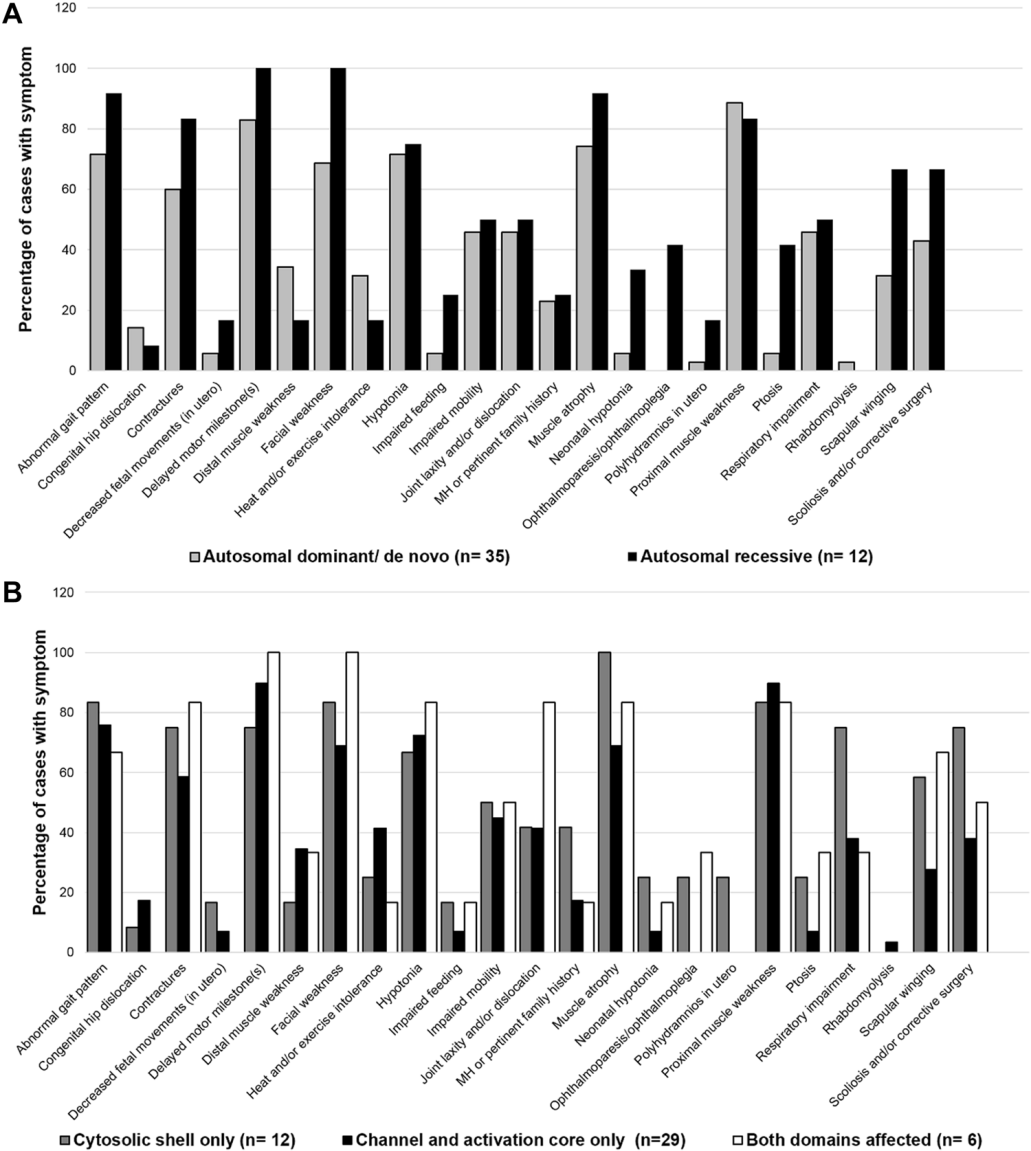
Bar charts of clinical symptom frequency by mode of inheritance (**a**) and affected RyR1 domain(s) (**b**) expressed as a percentage of cases. Statistically significant differences were as follows for AD/DN versus AR categories, respectively; facial weakness 69% versus 100%, *p* = 0.026; neonatal hypotonia 6% versus 33%, *p* = 0.034; ophthalmoplegia/paresis 0% versus 42%, *p* < 0.001; ptosis 6% versus 42%, *p* = 0.003; and scapular winging 31% versus 67%, *p* = 0.032. When symptom frequency was compared by affected RyR1 domain(s), the only statistically significant difference, after adjustment for multiple comparisons, was in ophthalmoplegia/paresis between CS versus CAC, 25% versus 0%, *p* = 0.005 and both domains versus CAC, 33% versus 0%, *p* = 0.001. Differences in symptom frequency for all other symptoms, by mode of inheritance and affected RyR1 structural domain(s), were not significant, *p* > 0.05

Clinical findings for each participant are provided in Table S1. The most frequently observed clinical manifestations in this cohort were delayed motor milestones and proximal skeletal muscle weakness (both observed in 87% of cases), followed by skeletal muscle atrophy (observed in 79% of cases), abnormal gait, and facial weakness (both observed in 77% of cases). In AR cases, facial weakness, neonatal hypotonia, ophthalmoplegia/paresis, ptosis, and scapular winging were more frequently observed than in AD/DN cases (all, *p* < 0.05), Fig. 3a. Of note, Ophthalmoplegia/paresis was only observed in AR cases (42%). In contrast, hypotonia, and delayed motor milestones were frequently observed regardless of the mode of inheritance (70–100% of cases, both *p* > 0.05). MHS or a pertinent family history for MH was evident in both AD/DN and AR cases (17% and 6% of cases, respectively, *p* > 0.05). Recurrent rhabdomyolysis was reported in a single dominant case with the proband and both male offspring all exhibiting exercise intolerance and myalgia.

### Structure-phenotype correlation

**Fig. 4:**
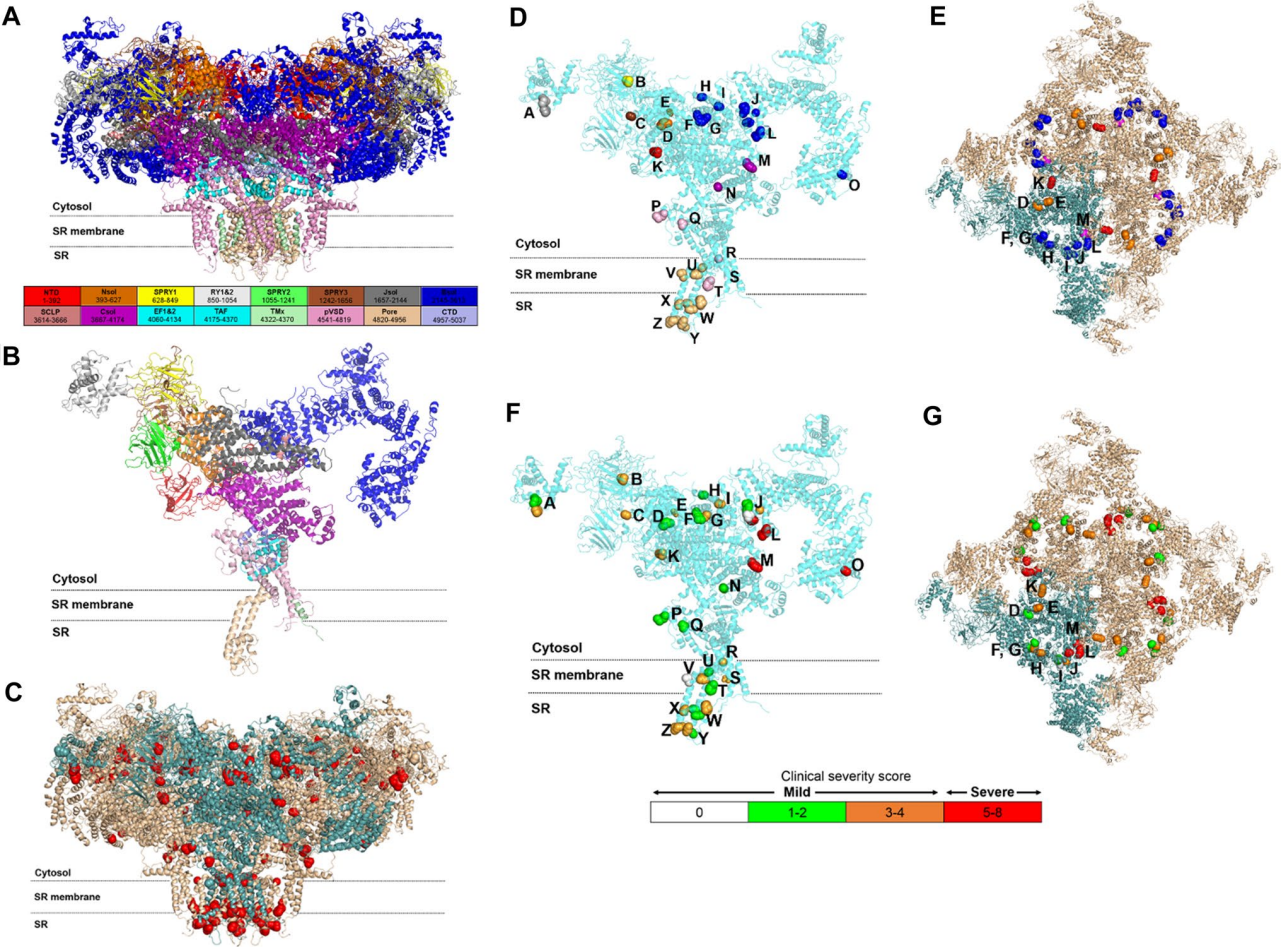
**a**–**g** Variants mapped to the high resolution cryo-EM mammalian (rabbit) RyR1 structure. Letters **a**–**g** correspond to specific, affected RyR1 residues. Lettering is consistent between panels. **a** Topographical image of the RyR1 tetrameric structure with each structural region assigned a unique color. **b** Topographical image of a single RyR1 monomer with each structural region assigned a unique color. **c** RyR1 protein structure is shown with a single tetrameric subunit highlighted in teal. Each variant is represented by a sphere which includes the whole side chain of the affected residue. Lettering **d**–**g** identify affected RyR1 residues: (A, p.Arg1043Cys/rabbit p.Arg1044; p.Arg975Trp/rabbit p.Arg976), (B, p.Asp708Asn/rabbit p.Asp709), (C, p.Arg1606His/rabbit p.Arg1607), (D, p.Arg530His/rabbit p.Arg531), (E, p.Met485Val/rabbit p.Leu486), (F, p.Arg2163His/rabbit Arg2163), (G, p.Thr2206Met/rabbit Thr2206), (H, p.Arg2224His/rabbit p.Arg2224), (I, p.Cys2233Arg/rabbit p.Cys2233), (J, p.Arg2336His/rabbit p.Arg2336; p.Asn2342Ser/rabbit p.Asn2342; p.Val2354del/rabbit p.Val2354; p.Gly2434Arg/rabbit p.Gly2434), (K, p.Arg109Trp/rabbit p.Arg110), (L, p.Arg2452Trp/rabbit p.Arg2452), (M, p.Tyr3933Cys/rabbit Tyr3934), (N, p.Ser4028Leu/rabbit p.Ser4029), (O, p.Arg3366/rabbit p.Arg3366), (P, p.Arg4737Gln/rabbit p.Arg4736), (Q, p.Thr4709Met/rabbit p.Thr4708), (R, p.Gly4820Arg/rabbit p.Gly4819), (S, p.Asn4575Thr/rabbit p.Asn4574), (T, p.Phe4808Asn/rabbit p.Phe4807), (U, p.Leu4936Arg/rabbit p.Leu4935; Ala4940Thr/rabbit p.Ala4939), (V, p.Met4840Arg/rabbit p.Met4839), (W, p.Arg4893Gln/rabbit p.Arg4892; p.Ala4894Asp/rabbit p.Ala4893; p.Ile4898Thr/rabbit p.Ile4897; p.Phe4921Leu/rabbit p.Phe4920), (X, p.Thr4853Ile/rabbit p.Thr4852), (Y, p.Met4875Val/rabbit p.Met4874; p.Glu4911Lys/rabbit p.Glu4910), (Z, p.Arg4861His/rabbit p.Arg4860). **d, e** Variants in each RyR1 region are assigned distinct colors, as detailed in **a, b. f, g** Variant mapping with color coding for clinical severity as follows: mild (clinically mild; 0 = white, 1–2 = green, 3–4 = orange. Clinically severe ≥ 5 = red). Clinical severity scores for each specific variant/participant are provided in Table S3. **e** RyR1 CS plane of interest with a single monomer highlighted in teal. View is facing the SR from the cytosol and variant coloring is as defined in **a, b**. Variants are enriched to the Bsol. **f** RyR1 CS plane of interest with a single monomer in teal. Clinical severity coloring, for each variant, is as defined in **c**. View is facing the SR from the cytosol. Variants with the greatest clinical severity are localized to the Bsol

Published functional assay results relating to specific variants are provided in Table 3. This table also includes details regarding whether variants are likely to impact an RyR1 functional site (e.g., triadin binding or inter-subunit interaction) and/or change amino acid composition, polarity, or molecular volume. Of the 46 coding region *RYR1* variants identified in this cohort, 24 affected the RyR1 CS domain and a further 22 affected the CAC domain. All missense substitution and deletion variants were mapped to the cryo-EM RyR1 structure, Fig. 4C-G. The evolutionary disparity between wild-type and mutant amino acids for missense substitution variants (*n* = 36), as determined by Grantham distance (5–215), ranged from 21 to 194 with a mean distance of 76. The two canonical splice site variants (c.8933-1 G > A and c.9001-2A > G) were located adjacent to exons 59 and 60, respectively, which contribute to encoding the bridging solenoid (Bsol) in the CS domain. A greater proportion of cases, with variant(s) affecting only the RyR1 CS, were clinically severe when compared to cases with variant(s) affecting only the RyR1 CAC (33% versus 7% respectively, *p* = 0.05), Fig. S3B. A breakdown of specific symptoms by affected RyR1 structural domain(s) is provided in Fig. 3B.

An AD/DN mode of inheritance was more frequently observed in participants with variant(s) that affected only the RyR1 CAC compared with only the CS (97% versus 50% of cases respectively, *p* < 0.001). In four cases (3, 5, 7, and 11), multiple variants were identified that affected only the RyR1 CS, (Table 3). Bsol was the most frequently affected region (83% of cases), in cases with only the RyR1 CS affected, followed by the RYR repeats 1 and 2 (RY1&2), (25% of cases). Only one case (34) had multiple *RYR1* variants that affected only the CAC. In cases with variants that affected only the CAC, the pore region inclusive of the helical-bundle between S2 and S3 (S2S3), was affected most often (76% of cases). In three related cases (39, 40, 41) with a rhabdomyolysis clinical phenotype, the same *RYR1* variant (p.Asp4505His) affected a currently unresolved region between amino acid residues 4354–4631 [64]. In cases with variants that affected both RyR1 domains, Bsol within the CS was the most frequently affected region (84% of cases).

**Fig. 5:**
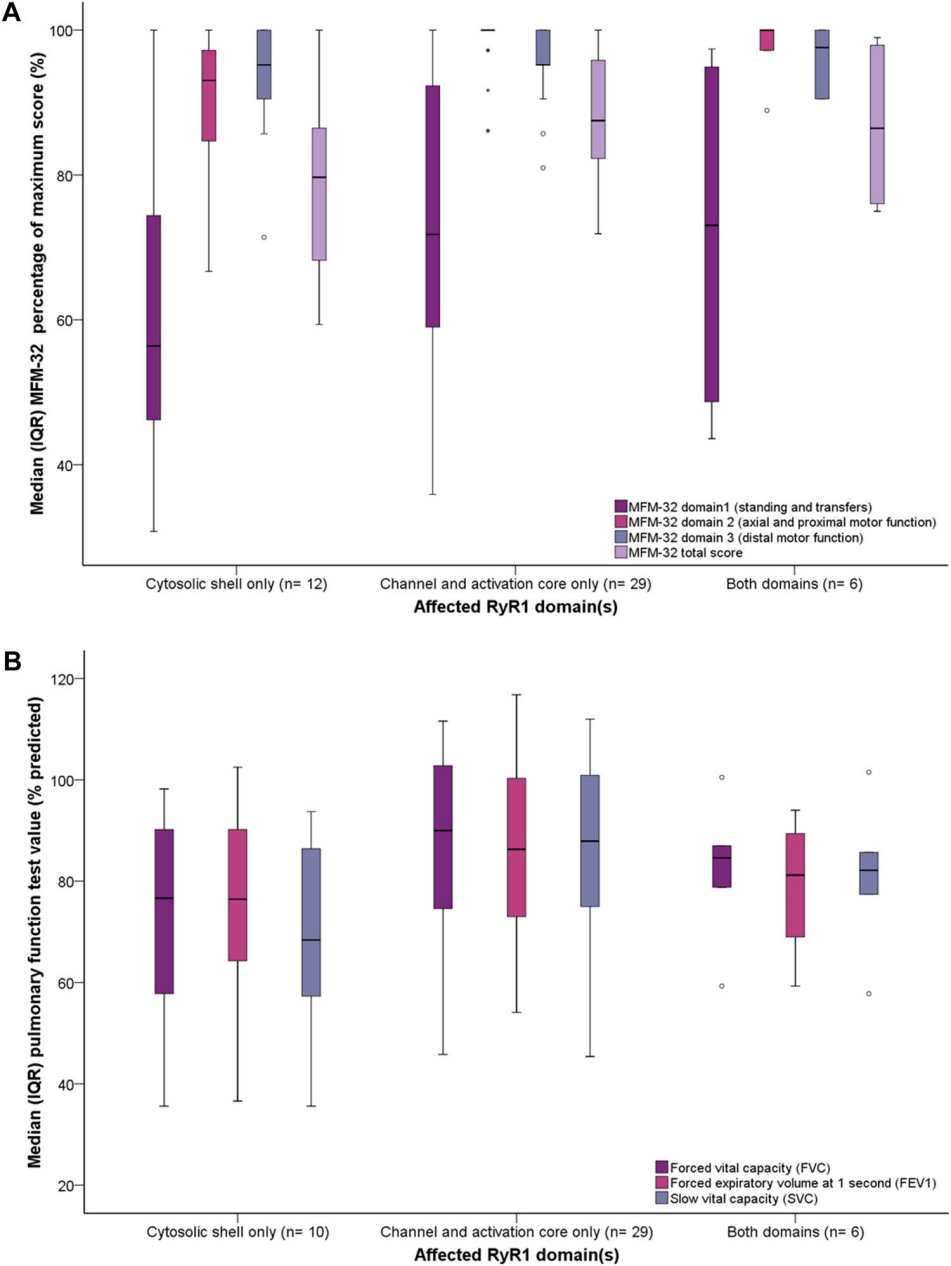
Median (IQR) MFM-32 scores and pulmonary function test values by affected RyR1 domain(s). A trend was observed for participants with only the CS affected, to exhibit lower median (IQR) percentage of maximum MFM-32 scores for each dimension including total score, **a**. This was deemed statistically significant for comparison of dimension 2 only (axial and proximal motor function), after adjustment for multiple comparisons (only the CS affected, 93.1 (13.2)% versus only the CAC affected 100.0 (1.4)% respectively, *p* < 0.001). There was no difference in percent predicted maximal effort PFTs according to affected RyR1 domain(s), **b**. Cases, with variant(s) that affected only the CS, achieved a significantly lower percent predicted SVC when compared to cases with variants that affected only the CAC, after adjustment for multiple comparisons (69.5 ± 17.3% versus 87.0 ± 18.0% respectively, *p* = 0.03), **b** (^∘^ and * denote outliers)

Cases with variants affecting only the CS had lower scores for MFM-32 dimension 2 (axial and proximal motor function), when compared to cases with only the CAC affected, after adjustment for multiple comparisons [93.1 (13.2)% versus 100 (1.4)% respectively, *p* < 0.001, Fig. 5a]. There was no significant difference in percent predicted maximal effort PFTs according to affected RyR1 domain(s), Fig. 5b. Yet cases, with variant(s) affecting only the RyR1 CS, achieved a significantly lower mean percent predicted SVC when compared to cases with variants affecting only the RyR1 CAC, after adjustment for multiple comparisons (69.5 ± 17.3% versus 87.0 ± 18.0% respectively, *p* = 0.03), Fig. 5b. A greater proportion of cases with only the RyR1 CS affected, exhibited moderate respiratory insufficiency compared to cases with only the RyR1 CAC affected (40% versus 3% respectively, *p* = 0.01), Fig. S5c–e. There were no other statistically significant differences between groups.

### Detailed variant mapping and analysis

Detailed *RYR1* variant mapping is provided with possible structural consequence in Figs. S6–S39. Variant map analysis demonstrated that, in this cohort, affected residues in cases with mild clinical severity (labelled white, green, and orange in Fig. 4f, g) were predominantly clustered to the CAC and the top portion of the CS. Clinically severe cases (labelled red in Fig. 4f, g) were limited to the CS. Affected residues at the interface of distinct RyR1 regions are detailed in Fig. S40.

In AR cases with premature termination or deletion variants (cases 3, 5, 10, 11, 12, 44, and 47) nonfunctional protein is coded. These variants likely result in decreased protein production, via nonsense-mediated decay of mutant mRNA, as supported by prior reports (see Table 3). In some cases, a single missense substitution that is considered likely pathogenic occurs in the same individual expressed on the other allele. Two individuals (cases 44 and 47) had termination variants which affected a residue in either the CS or CAC, as well as additional single or multiple missense substitution variants that affected the opposite RyR1 domain and were expressed on the other allele. In these cases, the termination variant likely results in decreased RyR1 expression with variants on the other allele exacerbating the individual’s phenotype via RyR1 dysfunction. Several other recessive cases had termination or deletion variants and/or multiple missense substitutions and/or a duplication variant (cases 5, 7, 42, 43, and 47). A detailed structure–function/phenotype review of these cases is provided within Supplementary Material.

Mapping of variants on the RyR1 tetramer revealed that many localized to one plane in the outermost portion of the cytosolic shell (Fig. 4e, g). Herein, this will be referred to as the CS plane of interest and includes residues from the NTD (residues 1–392), Nsol (residues 393–627), Bsol (residues 2145–3613) and Csol (residues 3667–4174). Disease-associated variants in the Bsol and Csol were flanked on one side by the locations of variants in Nsol of the same subunit and on the other side by the site of variants in the NTD of the neighboring subunit. Thus, the CS plane of interest highlighted in Fig. 4e, consists of both intramolecular and intermolecular interactions between different domains.

We further evaluated the clinical severity scores assigned to variants within the CS plane of interest. In the interacting Bsol, Nsol, and NTD regions, clinical severity was associated with Grantham distance, which ranged from > 100 for severe to ~ 20 for mild. Variants attributed to differing clinical severity (shown as red, orange and green in Fig. 4g) were distributed in clusters through the interdomain contact region, in an apparent gradient. Variants associated with a clinically severe phenotype were localized to Bsol in each subunit; those associated with an intermediate phenotype were found at the intermolecular contact between Nsol and NTD; and those associated with a mild phenotype were in intramolecular contacts between the Bsol and Nsol.

In this cohort, *RYR1* variants localized to the CAC along three horizontal planes (Fig. S41), each of which was associated with an average clinical severity score of 3. Herein, these will be referred to as CAC planes of interest 1, 2 and 3, respectively. All variants discussed in this domain were localized to inter-subunit contact regions. As such, variants form a ring and are cooperative. CAC plane of interest 1 included sites where luminal loops connect to the pore [variant (clinical severity score); Met4875Val (4), Glu4911Lys (2), Arg4861His (3), and Arg4893Gln (4)]. CAC plane of interest 2 lies in the Ca^2+^ entry pore [variant (clinical severity score); Ala4894Asp (4), Ile4898Thr (2), and Phe4921Leu (2)]. CAC plane of interest 3 is located where the SR membrane region transitions into the cytosol and includes major structural elements [variant (clinical severity score); Leu4936Arg (4), Ala4940Thr (2), Gly4820Arg (4), and Met4840Arg (0)].

## Discussion

Clinical manifestations encompassed by the *RYR1*-RD disease spectrum are notoriously diverse. Nevertheless, we corroborate genotype-phenotype correlations and, through variant mapping to the latest cryo-EM RyR1 structure, elucidate structural regions (CS plane of interest, and CAC planes of interest 1, 2, and 3) that may be important in determining clinical phenotype.

In this cohort, variants in AR cases were dispersed throughout the *RYR1* coding region; in accordance with prior reports [2, 93]. Due to the limited number of AR cases in this cohort, we cannot reject the null hypothesis that there is no difference in clinical severity by mode of inheritance. Nonetheless, the trend in this study is consistent with previous studies that report AR cases as being more clinically severe than AD/DN cases (25% versus 11%) [29, 30]. This may partially explain why, on average, AR cases were diagnosed over ten years earlier than AD/DN cases, in this cohort. In contrast to AR cases and as expected, *RYR1* variants inherited in an AD/DN manner were enriched within established MH/CCD hot spot regions, particularly MH/CCD hot spot 3 [89]. Variants affecting the C-terminal region, as defined by the latest cryo-EM RyR1 residue spans, were not identified in this cohort.

Massively parallel sequencing has been fundamental in achieving earlier *RYR1*-RD diagnoses. Indeed, study participants born before the advent of this technology in 2004 were typically diagnosed as adults whereas those born after 2004 were generally diagnosed in early childhood, which underscores the utility of high-throughput sequencing technologies for this rare disease [16]. The ability to achieve an early *RYR1*-RD diagnosis has been further aided by the development of multi-gene congenital myopathy panels which, due to greater availability and decreased cost, should be considered the standard approach when seeking to confirm a specific genetic etiology [54]. Although histopathologic features have been used to define *RYR1*-RD subtypes, these features are variable over time and there is considerable overlap among categories, such as MmD, CNM, and CRM [86]. Such overlap was evident in this study as 31% of participants with biopsy reports (*n* = 26) received inconclusive or non-specific histopathologic diagnoses. Our results further support a genetics-led diagnostic approach for congenital neuromuscular disorders.

The clinical findings in family 14 (cases 17–18, p.Ala4940Thr) and family 18 (cases 30–31, p.Ala4894Asp) reinforce the concept of variable expressivity in *RYR1*-RD. Indeed clinical manifestations were not consistent among family members despite having identical *RYR1* variants. This intrafamilial phenotypic variability in *RYR1*-RD highlights the importance of personalizing care plans, even among related individuals with an identical genetic etiology. Undetected variants and presence of genetic modifiers may contribute to intrafamilial phenotypic variability however parental mosaicism may also offer a potential explanation for inter-generational phenotypic variability within families [47]. Our clinical findings expand the clinical heterogeneity associated with the p.Ile4898Thr variant, previously associated with moderate, severe and lethal phenotypes. In this cohort, two related individuals with a comparably mild phenotype (clinical severity score of 2) expressed this variant.

In family 30, the mother reported a history of recurrent rhabdomyolysis which was accompanied by exercise intolerance and myalgia that both male offspring also exhibited. Of note, the p.Asp4505His variant has been previously implicated in a fatal non-pharmacologic induced MH episode, late-axial myopathy, and idiopathic hyperCKemia with MHS [20, 41, 44]. This reinforces the importance of detecting *RYR1* variants, even in those with only mild myopathy-related symptoms, as such individuals can still be at risk.

In this cohort, in utero and neonatal manifestations, with the exception of congenital hip dislocation, were associated with AR cases. In families with a medical history pertinent for congenital myopathy, this observation may become an important component of the *RYR1*-RD differential workup, by enabling families to prepare and clinical teams to counsel for an infant that could emerge to be on the severe end of the *RYR1*-RD spectrum of disease.

A greater proportion of cases, with variant(s) that affected only the RyR1 CS, were clinically severe when compared to cases with variant(s) that only affected the RyR1 CAC yet there was no difference in the proportion of clinically severe cases by mode of inheritance. These findings may reflect limitations of tools currently available to assess clinical severity in the *RYR1*-RD population. Focusing on two facets of the disease, ambulation and respiratory function [2], may lead to an underestimation of disease severity when additional factors such as feeding difficulties, eye involvement and degree of scoliosis also contribute. As such, development of a multifacted, validated clinical severity assessment tool for congenital neuromuscular disorders would undoubtedly strengthen future studies.

Strengths of this study include the use of robust measures of motor function (MFM-32), respiratory function (PFTs in accordance with ATS guidelines), and a single clinician administered physical examinations that minimized variability in participant reports. Non-ambulatory individuals and those < 7 years of age were excluded from clinical trial participation and this may have prevented our analysis from capturing the genotype/structure-phenotype of individuals at the most severe end of the *RYR1*-RD disease spectrum. Nonetheless, we demonstrate, for the first time using a validated tool, that *RYR1*-RD affected individuals have greatest difficulty performing movements that involve standing and transfers and that this deficit is comparable regardless of the mode of inheritance. Moreover, in this cohort, AR cases had the greatest motor function impairment overall, owing to additional difficulties performing axial, proximal, and distal movements. The identification of variable motor deficits, by mode of inheritance, suggests that tailored interventions would likely be appropriate in exercise/physical therapy-based clinical trials.

Cases with variants that affected only the RyR1 CS, achieved a significantly lower percent predicted SVC compared to cases with variants that affected only the CAC or both domains, indicating that variant location may, at least in part, dictate clinical phenotype. Those affected by neuromuscular disease often have difficulty sustaining the forced exhalation required for FVC and FEV_1_ measurement [32]. As such, SVC may provide a suitable alternative measure of respiratory function with adequate sensitivity to detect differences among *RYR1*-RD sub-groups. Furthermore, SVC may serve as a useful clinical trial endpoint, subject to successful validation studies.

Several variants with minor physico-chemical changes were still associated with an *RYR1*-RD clinical phenotype. This could indicate that such residues have a functional role or are important to nearby functional residues, such as p.Arg2163 being close to the proposed FKBP12 binding site (Fig. 4e, g, Letter F) and p.Ser4028 being nearby to the putative Ca^2+^ binding site (Fig. 4d, f, letter N) [13, 51, 65]. As a result, such residues may be less able to tolerate variations.

Structural regions included within the CS plane of interest (NTD, Nsol, Bsol, and Csol) are far apart in sequence, but close in 3D space in the context of the tetramer, allowing long-range cooperativity, consistent with the observation that variations in the CS can affect gating in the CAC [84]. Most variants in this plane fell within either MH/CCD hot spots 1 or 2 (*n* = 3 and *n* = 9, respectively). Many of these variants that have been functionally characterized in vitro (5/9) result in Ca^2+^ leakage, highlighting the functional importance of the CS in allosteric gating of the channel. The p.Tyr3933Cys substitution in the CS plane of interest is located in an inter-domain interaction between the CS and the CAC, suggesting it may be involved in transmitting the signal for pore gating. Many variants within the CS plane of interest occur at positions that are exposed to solution, often in open cavities, suggesting these cavities have important roles in channel function. Indeed, recent biophysical simulation studies have revealed additional ion conduction pathways that permeate the lateral aspect of the RyR1 CS and may thereby enable lateral Ca^2+^ efflux into the cytoplasm [22].

It is possible that variants within CAC plane of interest 1 affect Ca^2+^ flux or selectivity at the vestibule adjoining the entry pore. Variants within CAC plane of interest 2 likely affect entry pore function. p.Ala4894Asp alters the charge distribution, while p.Ile4898Thr and p.Phe4921Leu affect the pore structure itself thereby impairing Ca^2+^ selectivity or conductance [56]. Within CAC plane of interest 3, Leu4936 contributes to α helix-α helix packing and is immediately adjacent to Ile4937, a critical channel gating residue. Indeed, it is the hydrophobic properties of Ile4937, that enable this residue to form a physical gate at the narrowest section of the pore preventing Ca^2+^ flux in the RyR1 closed state [85]. All other variants within this plane occur at regions where sharp turns of the helices are formed. These regions are important for defining the structure and electrostatic properties of the gating regions.

## Conclusion

Our comprehensive analyses corroborate genotype-phenotype associations and identify new protein structure-phenotype correlations and structural planes of interest that warrant further investigation. Through structural assessment of patient-derived *RYR1* variants, we show that although both RyR1 domains function together to enable optimal SR Ca^2+^ efflux, variants affecting the CS were associated with a more severe clinical phenotype. In particular, variants within the CS plane of interest were enriched in the Bsol that is crucial for maintaining effective inter-subunit interactions and channel gating. We demonstrate that variant location likely dictates clinical severity, in combination with the mode of inheritance, and degree of physico-chemical disruption, at RyR1 regions sensitive to structural modification.

## Electronic supplementary material

Below is the link to the electronic supplementary material.

Supplementary material 1 (PDF 378 KB)

Supplementary material 2 (PDF 5088 KB)

Supplementary material 3 (PDF 2562 KB)
