# Supplementary material for: Correlation of phenotype with genotype and protein structure in RYR1-related disorders
Source: J Neurol. 2018 Aug 28;265(11):2506–24. doi: 10.1007/s00415-018-9033-2 (PMC6182665; doi:10.1007/s00415-018-9033-2)
Supplement: Supplementary file 1 — Supplementary material 1 (PDF 378 KB) [file 415_2018_9033_MOESM1_ESM.pdf]

Correlation of phenotype with genotype and protein structure in *RYR1*-related disorders

Supplementary Material

Joshua J. Todd [1*], Vatsala Sagar [2], Tokunbor A. Lawal [1], Carolyn Allen [1], Muslima S. Razaqyar [1], Monique S. Shelton [1], Irene C. Chrismer [1], Xuemin Zhang [1], Mary M. Cosgrove [1], Anna Kuo [1], Ruhi Vasavada [3], Mina S. Jain [3], Melissa Waite [3], Dinusha Rajapakse [2], Jessica W. Witherspoon [1], Graeme Wistow [2], Katherine G. Meilleur [1].

[1] Neuromuscular Symptoms Unit, National Institute of Nursing Research, National Institutes of Health, Bethesda, MD, United States

[2] Section on Molecular Structure and Functional Genomics, National Eye Institute, National Institutes of Health, Bethesda, MD, United States

[3] Mark O. Hatfield Clinical Research Center, Rehabilitation Medicine Department, National Institutes of Health, Bethesda MD, USA

Table S1. Clinical manifestations and motor function for each participant by affected RyR1 structural region

	Clinical symptoms																								Motor function			
**Case**	Abnormal gait pattern	Ambulatory	Congenital hip dislocation	Contractures	Decreased fetal movements in utero	Delayed motor milestones	Distal muscle weakness	Facial weakness	Heat and/or exercise intolerance	Hypotonia	Impaired feeding	Impaired mobility	Joint laxity or dislocation	MH episode or pertinent in family history	Muscle atrophy	Neonatal hypotonia	Ophthalmoparesis/ophthalmoplegia	Polyhydramnios in utero	Proximal muscle weakness	Ptosis	Respiratory impairment	Rhabdomyolysis	Scapular winging	Scoliosis (or prior surgery)	Standing and transfers (% maximum score)	Axial and proximal motor function (% maximum score)	Distal motor function (% maximum score)	Total MFM score (% maximum score)
**Participants with variant(s) affecting only the RyR1 cytosolic shell**																												
1	+	+	-	+	-	+	+	+	-	+	-	+	+	+	+	-	-	-	+	-	+	-	-	+	59.0	94.4	100.0	81.3
2	-	+	-	-	-	+	-	+	+	+	-	-	+	-	+	-	-	-	+	-	+	-	+	+	82.1	97.2	90.5	89.6
3	+	+	-	+	-	+	-	+	-	+	-	-	-	-	+	-	-	-	+	-	+	-	+	+	74.4	100.0	95.2	88.5
4	+	+	-	+	-	+	+	+	+	+	-	+	+	+	+	-	-	-	+	-	+	-	+	+	35.9	91.7	95.2	69.8
5	+	+	-	-	+	+	-	+	-	-	-	+	+	-	+	-	+	+	+	-	+	-	+	+	46.2	75.0	85.7	65.6
6	+	+	-	+	-	-	-	+	-	-	-	+	-	+	+	-	-	-	+	-	-	-	+	+	46.2	97.2	90.5	75.0
7	+	+	-	+	-	+	-	+	-	-	-	-	-	+	+	-	-	-	-	+	-	-	-	+	74.4	86.1	100.0	84.4
8	+	+	-	+	-	-	-	-	+	+	-	-	-	-	+	-	-	.	+	-	+	-	-	-	53.8	91.7	100.0	78.1
9	-	+	-	-	-	-	-	-	-	-	-	-	-	-	+	-	-	+	+	-	-	-	-	-	100.0	100.0	100.0	100.0
10	+	+	-	+	.	+	-	+	-	+	+	+	-	-	+	+	+	.	+	+	+	-	+	-	30.8	83.3	71.4	59.4
11	+	+	+	+	+	+	-	+	-	+	+	+	+	-	+	+	+	+	+	+	+	-	+	+	53.8	66.7	90.5	66.7
12	+	+	-	+	-	+	-	+	-	+	-	-	-	+	+	+	-	.	-	-	+	-	-	+	61.5	97.2	95.2	82.3
**Participants with variant(s) affecting only the RyR1 channel and activation core**																												
13	+	+	-	-	-	+	-	-	-	+	-	-	+	-	-	-	-	.	+	-	+	-	-	+	71.8	97.2	100.0	87.5

14	+	+	-	+	+	+	+	+	+	+	-	+	+	-	+	-	-	-	+	-	-	-	+	-	71.8	100.0	85.7	85.4
15	-	+	+	-	-	+	-	+	-	+	-	+	+	-	+	+	-	-	+	-	-	-	-	-	66.7	100.0	100.0	86.5
16	+	+	-	-	-	+	-	-	+	+	-	-	+	-	+	-	-	-	+	-	-	-	-	+	97.4	100.0	100.0	99.0
17	+	+	-	+	-	+	-	+	+	+	+	+	+	-	-	-	-	-	+	-	-	-	-	-	51.3	100.0	95.2	79.2
18	-	+	-	-	-	+	-	-	-	+	-	-	-	-	+	-	-	-	+	-	-	-	-	+	94.9	100.0	100.0	97.9
19	-	+	+	+	-	+	-	+	-	+	-	+	+	+	+	-	-	-	+	-	+	-	-	-	53.8	86.1	100.0	76.0
20	+	+	-	+	-	.	-	-	+	-	-	-	-	+	+	-	-	-	+	-	+	-	+	-	97.4	100.0	100.0	99.0
21	+	+	-	-	-	+	+	+	+	+	-	-	-	+	+	-	-	-	+	-	+	-	+	+	92.3	100.0	90.5	94.8
22	+	+	-	-	-	+	+	-	+	+	-	-	-	+	+	-	-	-	+	-	+	-	+	+	89.7	100.0	95.2	94.8
23	+	+	-	+	-	+	+	-	-	+	-	+	-	-	+	-	-	-	+	-	-	-	-	-	59.0	100.0	100.0	83.3
24	+	+	+	+	-	+	-	+	-	+	-	+	-	-	-	-	-	-	+	-	-	-	-	+	56.4	100.0	95.2	81.3
25	+	+	.	+	.	+	-	+	-	+	+	+	-	-	+	.	-	.	+	+	+	-	-	+	59.0	86.1	95.2	77.1
26	+	+	-	+	-	+	-	+	-	+	-	-	+	-	+	-	-	-	+	-	+	-	-	.	61.5	100.0	95.2	83.3
27	+	+	.	+	.	+	-	+	-	+	-	+	+	-	+	.	-	.	+	-	+	-	-	-	59.0	100.0	95.2	82.3
28	+	+	.	+	.	+	-	+	-	-	-	-	-	-	+	.	-	.	+	+	-	-	-	-	35.9	97.2	95.2	71.9
29	-	+	-	-	.	.	-	+	-	-	-	-	-	-	-	-	-	.	+	-	+	-	-	-	89.7	100.0	100.0	95.8
30	+	+	+	+	-	+	-	+	+	+	-	+	-	-	+	-	-	-	+	-	-	-	+	+	89.7	100.0	90.5	93.8
31	+	+	-	+	-	-	-	+	-	-	-	+	-	-	+	-	-	-	-	-	-	-	-	-	92.3	100.0	95.2	95.8
32	+	+	-	+	-	+	+	-	+	+	-	-	-	-	+	-	-	-	+	-	+	-	+	+	53.8	91.7	95.2	77.1
33	+	+	-	-	-	+	+	+	-	+	-	+	+	-	+	-	-	-	+	-	-	-	+	-	92.3	100.0	100.0	96.9
34	+	+	-	+	-	+	-	+	+	-	-	-	-	+	+	-	-	-	+	-	-	-	-	+	66.7	97.2	90.5	83.3
35	+	+	-	-	+	+	-	+	-	+	-	-	+	-	+	-	-	-	+	-	-	-	-	-	79.5	100.0	95.2	90.6
36	+	+	-	+	-	+	+	+	-	-	-	+	+	-	-	-	-	-	+	-	+	-	+	+	64.1	86.1	95.2	79.2
37	+	+	+	+	.	+	+	-	-	+	-	+	-	-	+	+	-	-	+	-	-	-	-	-	66.7	100.0	100.0	86.5
38	+	+	-	-	-	+	-	+	-	+	-	-	+	-	-	-	-	.	-	-	-	-	-	-	94.9	100.0	100.0	97.9
39	-	+	-	-	-	+	+	+	+	-	-	-	-	-	-	-	-	-	+	-	-	+	-	-	97.4	100.0	100.0	99.0
40	-	+	-	-	-	+	+	+	+	+	-	-	-	-	-	-	-	-	+	-	-	-	-	-	82.1	100.0	81.0	88.5
41	-	+	-	+	-	+	-	-	+	-	-	-	-	-	-	-	-	-	-	-	-	-	-	-	100.0	100.0	100.0	100.0

Participants with variant(s) affecting the RyR1 cytosolic shell and channel and activation core

42	-	+	-	+	-	+	-	+	-	+	-	+	+	-	+	-	-	-	+	+	+	-	+	+	56.4	88.9	95.2	77.1
43	+	+	-	+	-	+	-	+	-	+	+	+	+	-	+	+	-	-	+	-	-	-	+	-	43.6	100.0	90.5	75.0
44	+	+	-	+	-	+	+	+	+	+	-	-	+	-	+	-	+	-	+	-	-	-	+	-	97.4	100.0	100.0	99.0
45	-	+	-	+	-	+	-	+	-	-	-	-	+	+	+	-	-	-	-	-	+	-	-	+	94.9	100.0	100.0	97.9
46	+	+	-	+	-	+	-	+	-	+	-	+	-	-	+	-	-	-	+	+	-	-	+	+	48.7	97.2	90.5	76.0
47	+	+	-	-	-	+	+	+	-	+	-	-	+	-	-	-	+	-	+	-	-	-	-	-	89.7	100.0	100.0	95.8

Table S2. Clinical severity score for *RYR1* variants included in Figure 4

Figure 5 annotation	Amino acid change (human residue number)	Equivalent amino acid (rabbit residue number)	Clinical severity score and grading [a]	Clinical severity score range (if applicable)
A	p.Arg1043Cys	p.Arg1044	2 (clinically mild)	
A	p.Arg975Trp	p.Arg976	4 (clinically mild) [a]	2-6
B	p.Asp708Asn	p.Asp709	4 (clinically mild)	
C	p.Arg1606His	p.Asn1607	3 (clinically mild)	
D	p.Arg530His	p.Arg531	2 (clinically mild)	
E	p.Met485Val	p.Leu486	4 (clinically mild)	
F	p.Arg2163His	p.Arg2163	2 (clinically mild)	
G	p.Thr2206Met	p.Thr2206	4 (clinically mild)	
H	p.Arg2224His	p.Arg2224	2 (clinically mild)	
I	p.Cys2233Arg	p.Cys2233	4 (clinically mild)	
J	p.Arg2336His	p.Arg2336	2 (clinically mild)	
J	p.Asn2342Ser	p.Asn2342	0 (clinically mild)	
J	p.Val2354del	p.Val2354	7 (clinically severe)	
J	p.Gly2434Arg	p.Gly2434	4 (clinically mild)	
K	p.Arg109Trp	p.Arg110	4 (clinically mild)	
L	p.Arg2452Trp	p.Arg2452	5 (clinically severe) [a]	4-6
M	p.Tyr3933Cys	p.Tyr3934	5 (clinically severe)	
N	p.Ser4028Leu	p.Ser4029	2 (clinically mild)	
O	p.Arg3366His	p.Arg3366	5 (clinically severe)	
P	p.Arg4737Gln	p.Arg4736	2 (clinically mild)	
Q	p.Thr4709Met	p.Thr4708	4 (clinically mild)	
R	p.Gly4820Arg	p.Gly4819	4 (clinically mild) [a]	4-5
S	p.Asn4575Thr	p.Asn4574	4 (clinically mild)	
T	p.Phe4808Asn	p.Phe4807	2 (clinically mild)	
U	p.Leu4936Arg	p.Leu4935	4 (clinically mild)	
U	p.Ala4940Thr	p.Ala4939	2 (clinically mild) [a]	
V	p.Met4840Arg	p.Met4839	0 (clinically mild)	
W	p.Arg4893Gln	p.Arg4892	4 (clinically mild) [a]	3-5
W	p.Ala4894Asp	p.Ala4893	4 (clinically mild) [a]	3-4
W	p.Ile4898Thr	p.Ile4897	2 (clinically mild)	
W	p.Phe4921Leu	p.Phe4920	2 (clinically mild)	
X	p.Thr4853Ile	p.Thr4852	4 (clinically mild)	
Y	p.Met4875Val	p.Met4874	4 (clinically mild)	
Y	p.Glu4911Lys	p.Glu4910	4 (clinically mild) [a]	2-5
Z	p.Arg4861His	p.Arg4860	3 (clinically mild) [a]	2-4

[a] clinical severity score was averaged as the same *RYR1* variant was identified in multiple participants

Structure-function implications in recessive cases

In recessive cases with premature termination or deletion variations (cases 3, 5, 10, 11, 12, 44, and 47) nonfunctional protein is coded. These variants likely result in decreased RyR1 expression, via nonsense-mediated decay of mRNA, as supported by prior reports, Table 3. In some cases, a single missense substitution that is likely to be deleterious occurs in the same individual, expressed on the other allele.

Several other compound heterozygous cases had termination or deletion variations and/or multiple missense substitutions and/or a duplication variation (Cases 5, 7, 42, 43, and 47):

Case 5 (p.Arg2241* + p.Arg109Trp + p.Asp708Asn + p.Met485Val): Clinical severity score of 4. The p.Arg109Trp (rabbit p.Arg110) is categorized by ACMG criteria as likely pathogenic. This is further supported by a Grantham distance of 101 and the localization of this variant to the cytosolic plane of interest (letter K in Figure 4D-E). The Grantham distance of the p.Asp708Asn (rabbit p.Asp709) variant of uncertain significance was only 23. Nevertheless, the location of this variant, within an FKBP interaction site, may explain this phenotype. The Met485Val (rabbit p.Leu486) is a SNP that is categorized as a variant of uncertain significance. Although localized within the cytosolic plane of interest (letter E in figure 4D-E); this variant affects a residue that is not conserved evolutionarily (Figure S1) so this variant is the least likely of the four to be pathogenic.

Case 7 (p.Arg530His + p.Arg1043Cys + p.Arg2336His). Clinical severity of 2. All variants identified in Case 7 were categorized as variants of uncertain significance. The p.Arg530His (rabbit p.Arg531) and Arg2336His (equivalent in rabbit) variants, both with a Grantham distance of 29, were localized to the cytosolic plane of interest (letters D and I in Figure 4D-E, respectively). The combination of a high Grantham distance (180) and being located at a site of an inter-RyR1 interaction makes it plausible that the p.Arg1043Cys (rabbit p.Arg1044) is the variant driving this participant's phenotype (letter A in Figure 4D-E).

Case 42 (p.Asn2342Ser + p.Met4840Arg). Clinical severity of 0. Both variants identified in this individual were classified as variants of uncertain significance. The Asn2342Ser (equivalent in rabbit) may be functionally consequential despite its Grantham distance of 46 owing to localization within the cytosolic plane of interest (letter J in Figure 4D-E). Similarly, the Met4840Arg **(**rabbit p.Met4839**)** variant may impact channel function due to a Grantham distance of 91 and close proximity to a sharp turn in a channel and activation core α helix, a key structural element.

Case 43 (p.Glu4911Lys + p.Ile1571Val + p.Arg3366His + p.Tyr3933Cys). Clinical severity of 5. The clinical severity of the p.Glu4911Lys (rabbit p.Glu4910) was 2, when expressed alone (Case 38 supporting a contributory role of the other three variants in determining clinical severity. Both Ile1571Val and Arg3366His had a Grantham distance of 29 and were localized to currently unresolved regions of the RyR1 structure and could therefore not analyzed structurally. The Tyr3933Cys variant had a high Grantham distance of 194 and this residue likely participates in intramolecular stacked π orbital interactions which cannot occur with the mutant Cys. It is likely that p.Glu4911Lys and p.Tyr3933Cys are the two variants driving this participant's severe clinical phenotype.

Case 47 (p.Glu4167* + p.Gly2434Arg + p.Met4875Val). Clinical severity of 4. The p.Gly2434Arg (rabbit p.Gly2435) variant has Grantham distance of 125 and is categorized as pathogenic. Moreover, this variant is localized to the cytosolic plane of interest (letter J in Figure 4D-E). The p.Met4875Val (rabbit p.Met4874), classified as a variant of uncertain significance, was localized to the pore region of the channel and activation core domain. Met4875 is at the end of four negatively charged luminal loops in the rabbit structure containing Asp4873 (human Asp 4873), Asp4870 (human Asp 4870), Glu4869 (human Glu4869), and Glu4867 (human 4867) framing the entrance of the pore itself. Affected residues in this region may affect luminal triadin binding as well as retention of RyR-CSQ proximity and ability for rapid Ca^{2+} release [1, 2]. The location of this variant in a critical structural region, suggests that p.Met4875Val may contribute to the participant's phenotype.

1 Lee CS, Hanna AD, Wang H, Dagnino-Acosta A, Joshi AD, Knoblauch M, Xia Y, Georgiou DK, Xu J, Long Cet al (2017) A chemical chaperone improves muscle function in mice with a RyR1 mutation. Nature communications 8: 14659 Doi 10.1038/ncomms14659

2 Lee JM, Rho SH, Shin DW, Cho C, Park WJ, Eom SH, Ma J, Kim DH (2004) Negatively charged amino acids within the intraluminal loop of ryanodine receptor are involved in the interaction with triadin. The Journal of biological chemistry 279: 6994-7000 Doi 10.1074/jbc.M312446200
