# Supplementary material for: Correlation of phenotype with genotype and protein structure in RYR1-related disorders
Source: J Neurol. 2018 Aug 28;265(11):2506–24. doi: 10.1007/s00415-018-9033-2 (PMC6182665; doi:10.1007/s00415-018-9033-2)
Supplement: Supplementary file 2 — Supplementary material 2 (PDF 5088 KB) [file 415_2018_9033_MOESM2_ESM.pdf]

**Correlation of phenotype with genotype and protein structure in *RYR1*-related disorders**

Supplementary Figures (1)

Joshua J. Todd [1*], Vatsala Sagar [2], Tokunbor A. Lawal [1], Carolyn Allen [1], Muslima S. Razaqyar [1], Monique S. Shelton [1], Irene C. Chrismer [1], Xuemin Zhang [1], Mary M. Cosgrove [1], Anna Kuo [1], Ruhi Vasavada [3], Mina S. Jain [3], Melissa Waite [3], Dinusha Rajapakse [2], Jessica W. Witherspoon [1], Graeme Wistow [2], Katherine G. Meilleur [1].

[1] Neuromuscular Symptoms Unit, National Institute of Nursing Research, National Institutes of Health, Bethesda, MD, United States

[2] Section on Molecular Structure and Functional Genomics, National Eye Institute, National Institutes of Health, Bethesda, MD, United States

[3] Mark O. Hatfield Clinical Research Center, Rehabilitation Medicine Department, National Institutes of Health, Bethesda MD, USA

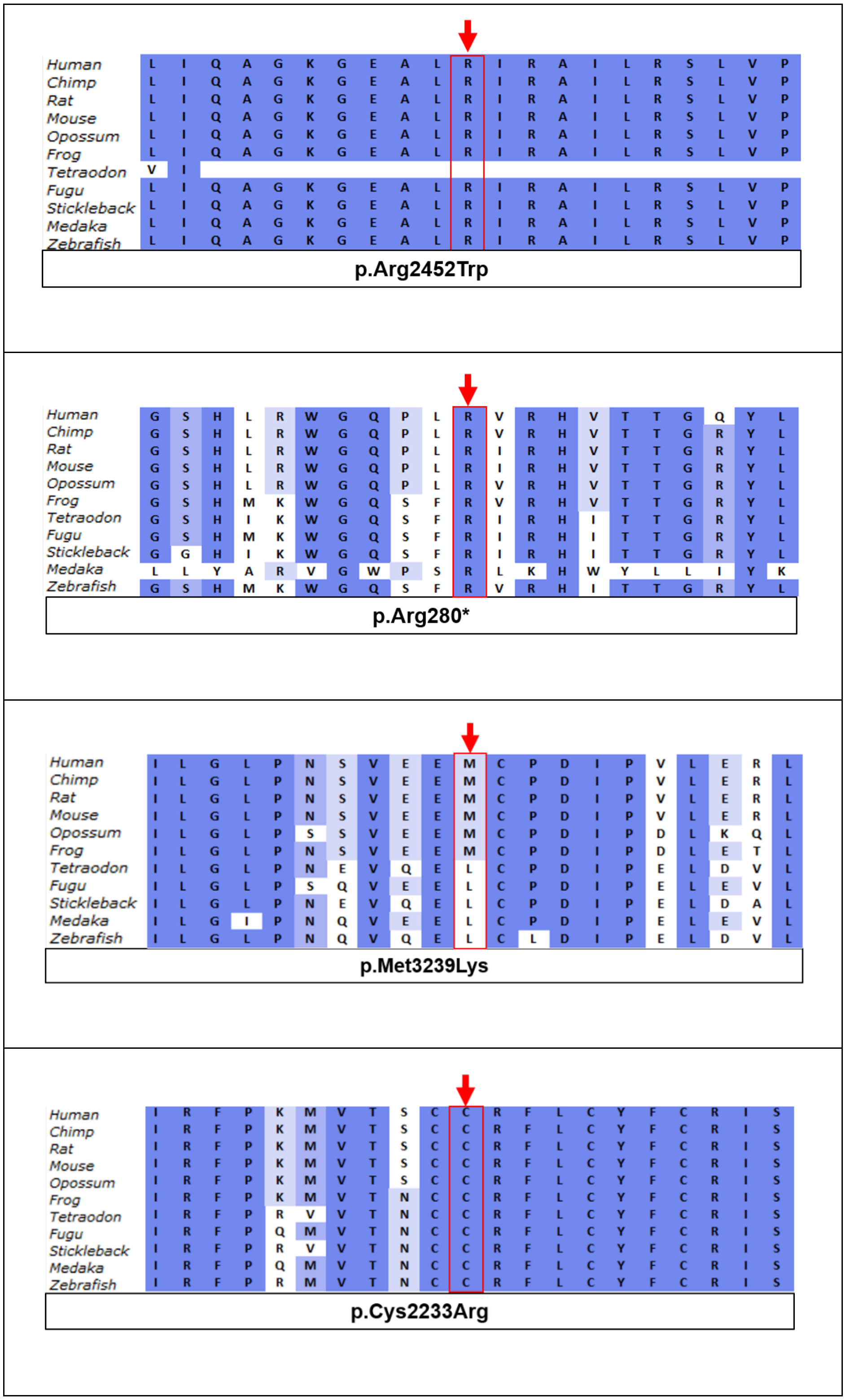
Human
Chimp
Rat
Mouse
Opossum
Frog
Tetraodon
Fugu
Stickleback
Medaka
Zebrafish
p.Arg2452Trp
p.Arg280*
p.Met3239Lys
p.Cys2233Arg

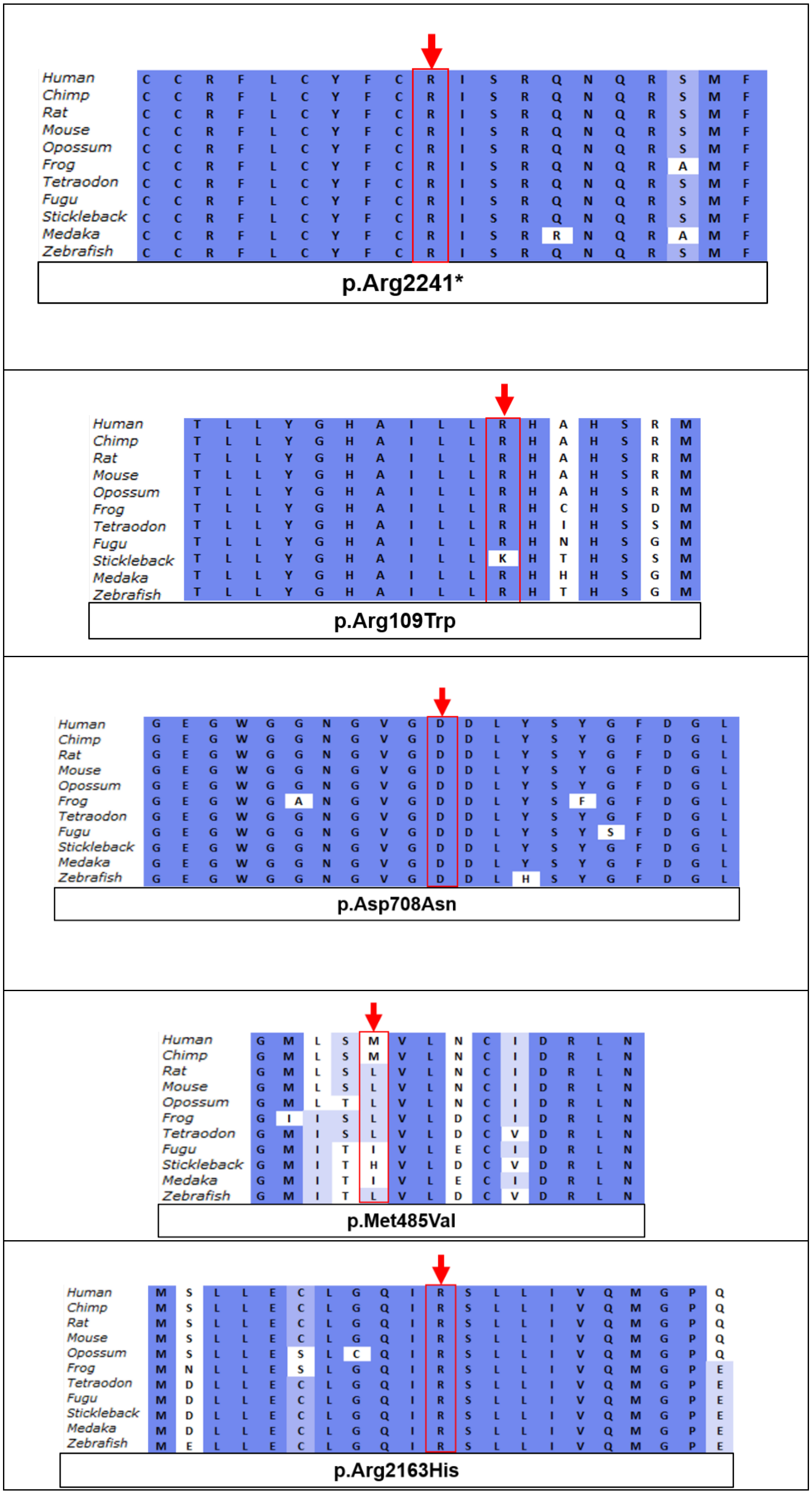
Human CCRFLCYFCRISRQNQRSMF
Chimp CCRFLCYFCRISRQNQRSMF
Rat CCRFLCYFCRISRQNQRSMF
Mouse CCRFLCYFCRISRQNQRSMF
Opossum CCRFLCYFCRISRQNQRSMF
Frog CCRFLCYFCRISRQNQRAMF
Tetraodon CCRFLCYFCRISRQNQRSMF
Fugu CCRFLCYFCRISRQNQRSMF
Stickleback CCRFLCYFCRISRQNQRSMF
Medaka CCRFLCYFCRISRRNQRAMF
Zebrafish CCRFLCYFCRISRQNQRSMF
p.Arg2241*
Human TLLYGHAILLRHAHSRM
Chimp TLLYGHAILLRHAHSRM
Rat TLLYGHAILLRHAHSRM
Mouse TLLYGHAILLRHAHSRM
Opossum TLLYGHAILLRHAHSRM
Frog TLLYGHAILLRHCHSDM
Tetraodon TLLYGHAILLRHIHSSM
Fugu TLLYGHAILLRHNHSGM
Stickleback TLLYGHAILLKHTHSSM
Medaka TLLYGHAILLRHHHSGM
Zebrafish TLLYGHAILLRHTHSGM
p.Arg109Trp
Human GEGWGGNGVGDDLYSYGFDGL
Chimp GEGWGGNGVGDDLYSYGFDGL
Rat GEGWGGNGVGDDLYSYGFDGL
Mouse GEGWGGNGVGDDLYSYGFDGL
Opossum GEGWGGNGVGDDLYSYGFDGL
Frog GEGWGANGVGDDLYSFGFDGL
Tetraodon GEGWGGNGVGDDLYSYGFDGL
Fugu GEGWGGNGVGDDLYSYSFDGL
Stickleback GEGWGGNGVGDDLYSYGFDGL
Medaka GEGWGGNGVGDDLYSYGFDGL
Zebrafish GEGWGGNGVGDDLHSYGFDGL
p.Asp708Asn
Human GMLSMVLNCIDRLN
Chimp GMLSMVLNCIDRLN
Rat GMLSLVLNCIDRLN
Mouse GMLSLVLNCIDRLN
Opossum GMLTLVLNCIDRLN
Frog GIISLVLDCIDRLN
Tetraodon GMISLVLDCVDRLN
Fugu GMITIVLECIDRLN
Stickleback GMITHVLDCVDRLN
Medaka GMITIVLECIDRLN
Zebrafish GMITLVLDCVDRLN
p.Met485Val
Human MSLLECLGQIRSLLIVQMGPQ
Chimp MSLLECLGQIRSLLIVQMGPQ
Rat MSLLECLGQIRSLLIVQMGPQ
Mouse MSLLECLGQIRSLLIVQMGPQ
Opossum MSLLESLCQIRSLLIVQMGPQ
Frog MNLLESLGQIRSLLIVQMGPE
Tetraodon MDLLECLGQIRSLLIVQMGPE
Fugu MDLLECLGQIRSLLIVQMGPE
Stickleback MDLLECLGQIRSLLIVQMGPE
Medaka MDLLECLGQIRSLLIVQMGPE
Zebrafish MELLECLGQIRSLLIVQMGPE
p.Arg2163His

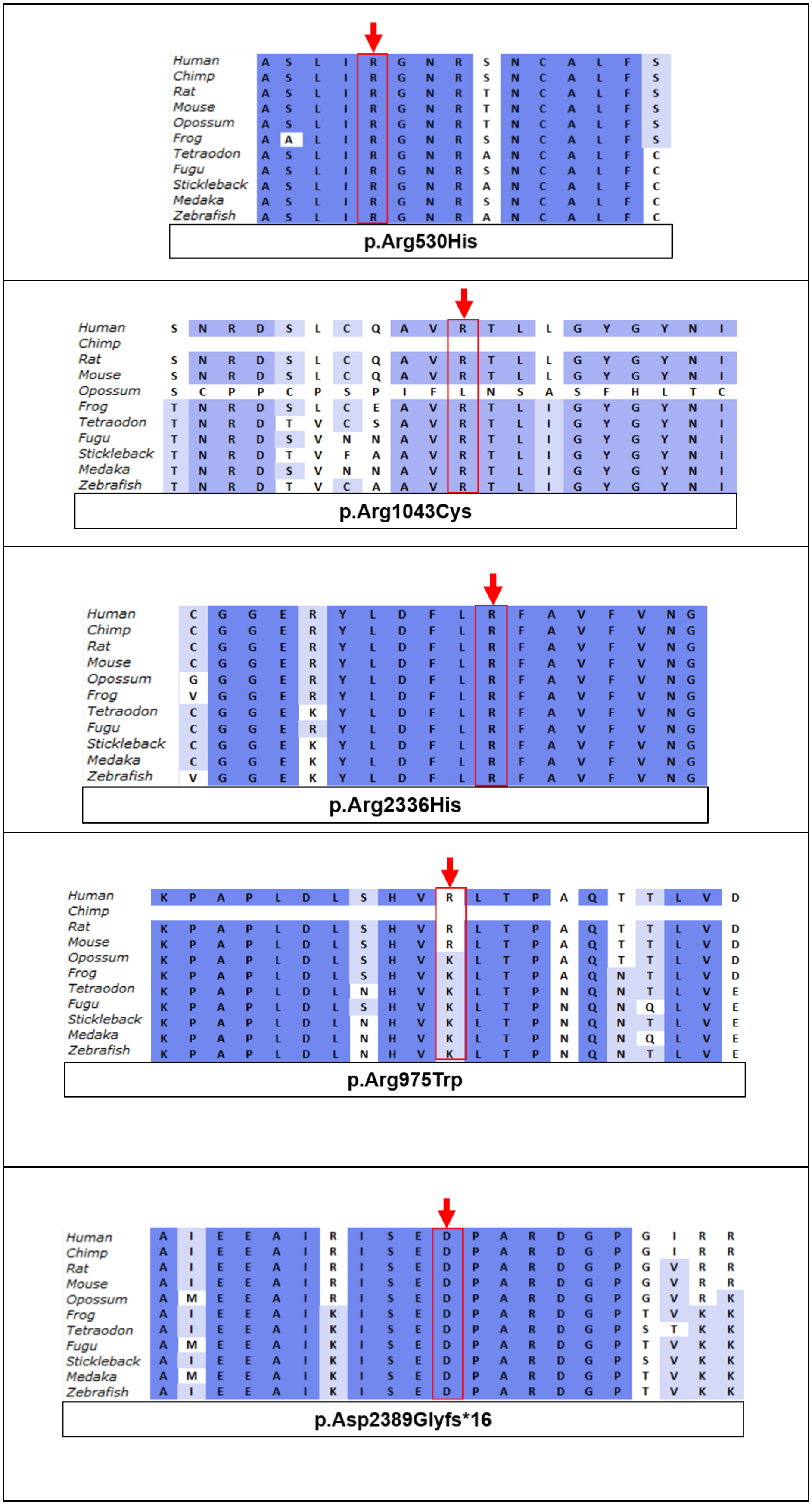
Human
Chimp
Rat
Mouse
Opossum
Frog
Tetraodon
Fugu
Stickleback
Medaka
Zebrafish
p.Arg530His
p.Arg1043Cys
p.Arg2336His
p.Arg975Trp
p.Asp2389Glyfs*16

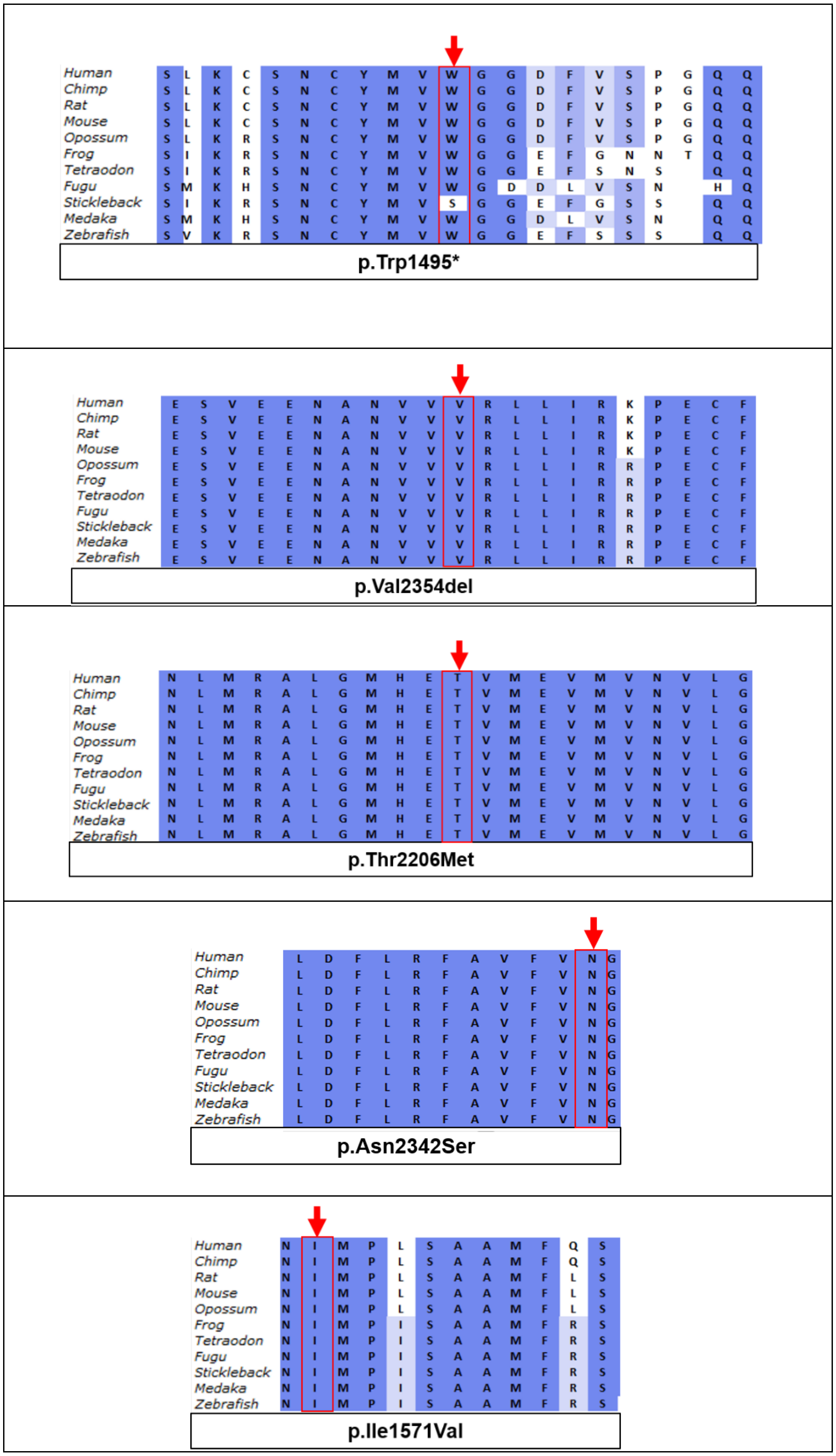
Human
Chimp
Rat
Mouse
Opossum
Frog
Tetraodon
Fugu
Stickleback
Medaka
Zebrafish
p.Trp1495*
p.Val2354del
p.Thr2206Met
p.Asn2342Ser
p.Ile1571Val

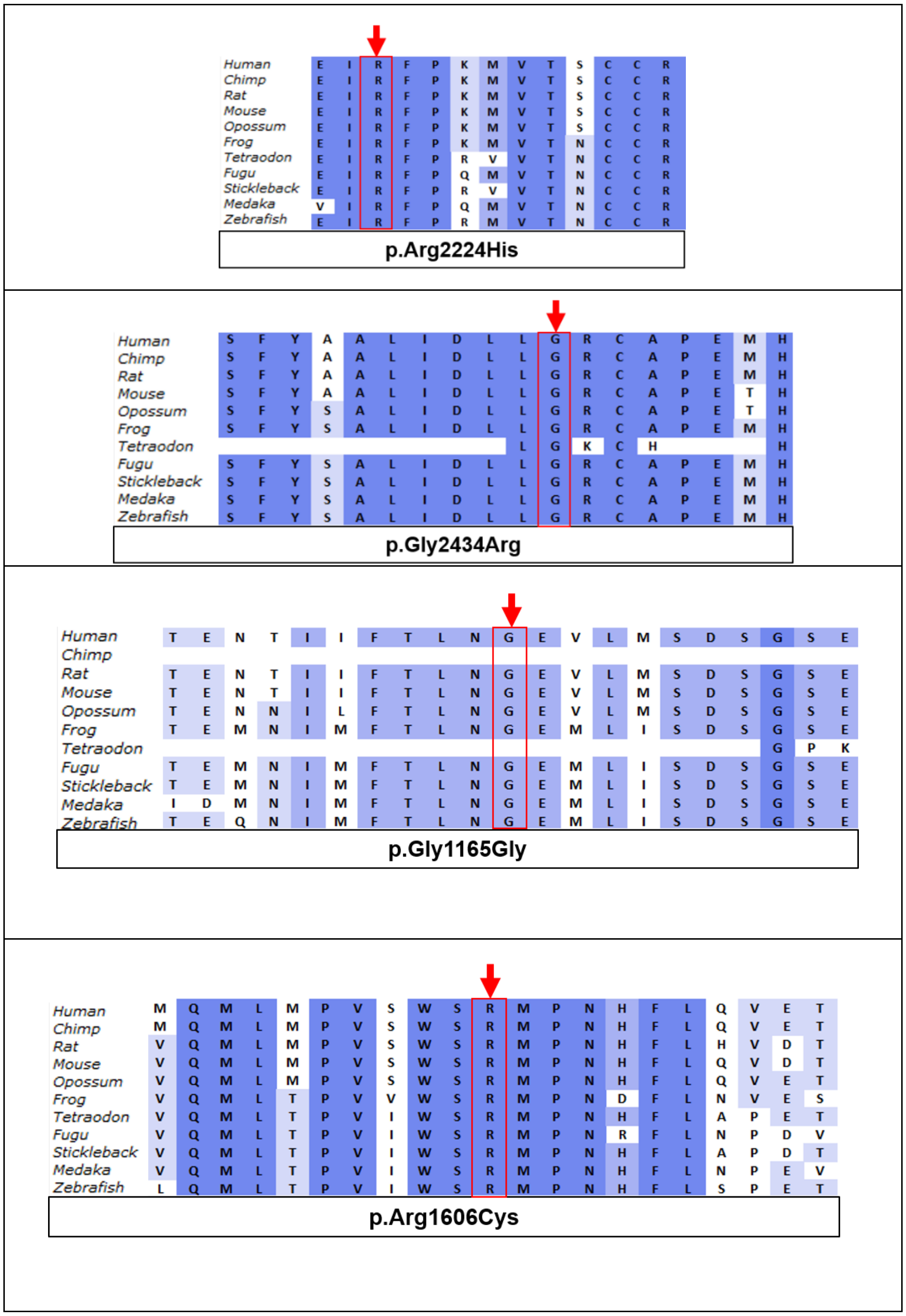


**Figure S1.** Orthologue alignments for each *RYR1* variant affecting the RyR1 cytosolic shell domain. With the exception of p.Met485Val and p.Arg1043Cys, all affected amino acid residues are highly conserved evolutionarily.

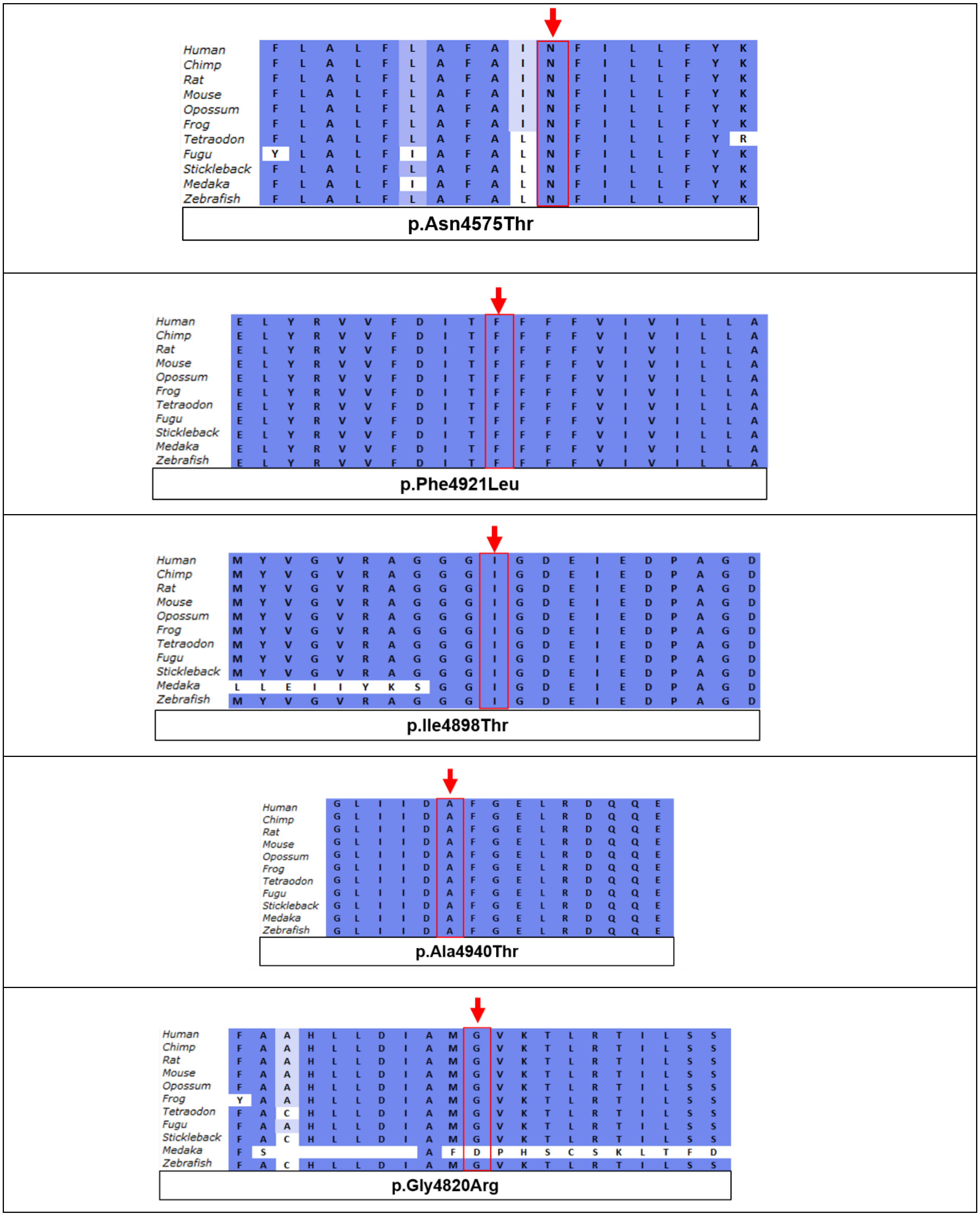
Human
Chimp
Rat
Mouse
Opossum
Frog
Tetraodon
Fugu
Stickleback
Medaka
Zebrafish
p.Asn4575Thr
p.Phe4921Leu
p.Ile4898Thr
p.Ala4940Thr
p.Gly4820Arg

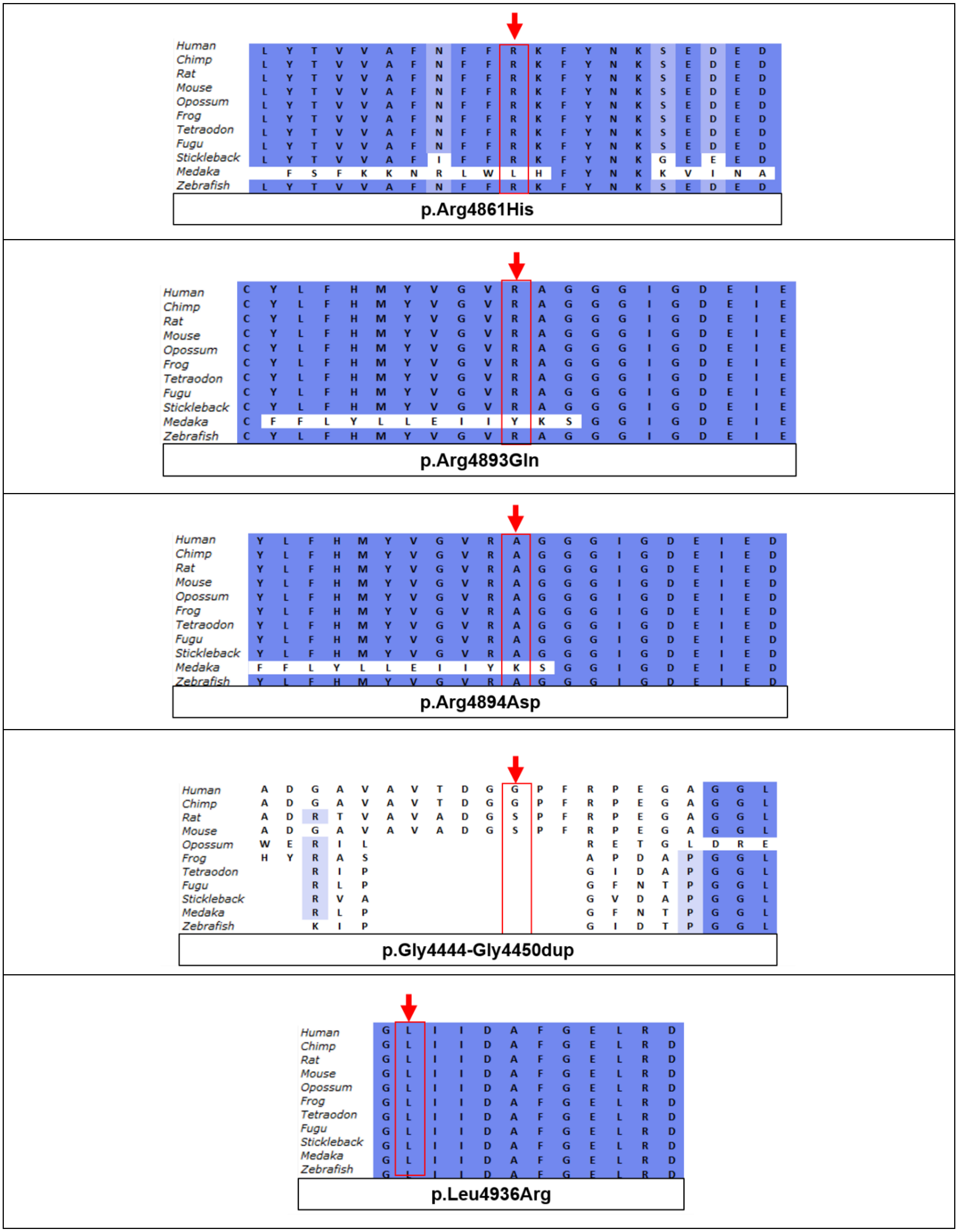
Human
Chimp
Rat
Mouse
Opossum
Frog
Tetraodon
Fugu
Stickleback
Medaka
Zebrafish
p.Arg4861His
p.Arg4893Gln
p.Arg4894Asp
p.Gly4444-Gly4450dup
p.Leu4936Arg

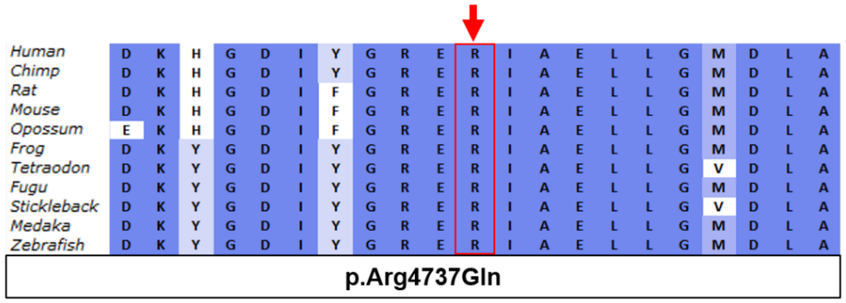
Human
Chimp
Rat
Mouse
Opossum
Frog
Tetraodon
Fugu
Stickleback
Medaka
Zebrafish
p.Arg4737Gln

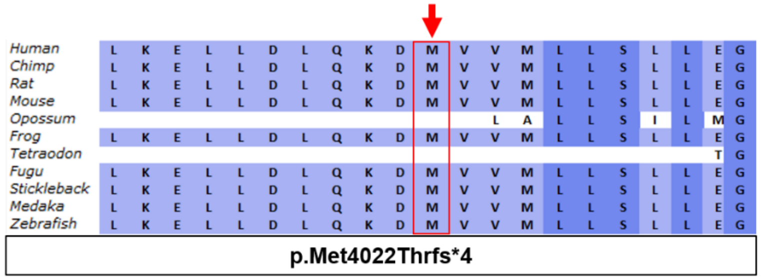
Human
Chimp
Rat
Mouse
Opossum
Frog
Tetraodon
Fugu
Stickleback
Medaka
Zebrafish
p.Met4022Thrfs*4

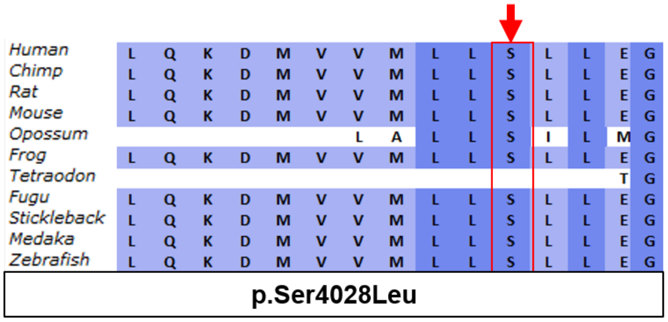
Human
Chimp
Rat
Mouse
Opossum
Frog
Tetraodon
Fugu
Stickleback
Medaka
Zebrafish
p.Ser4028Leu

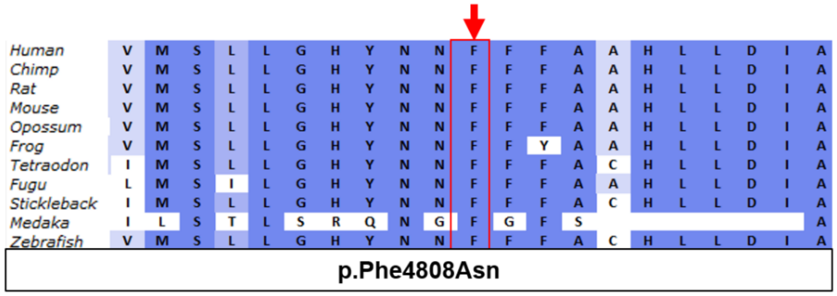
Human
Chimp
Rat
Mouse
Opossum
Frog
Tetraodon
Fugu
Stickleback
Medaka
Zebrafish
p.Phe4808Asn

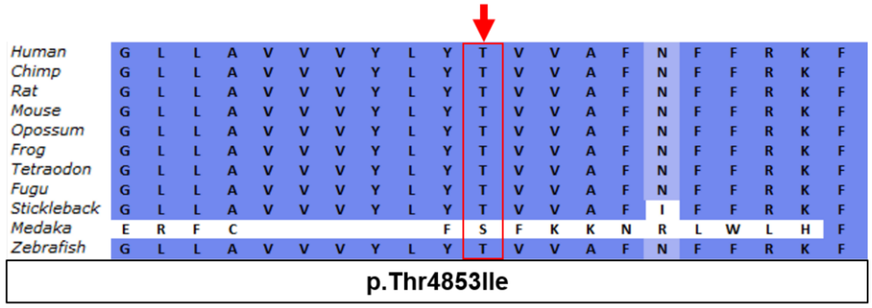
Human
Chimp
Rat
Mouse
Opossum
Frog
Tetraodon
Fugu
Stickleback
Medaka
Zebrafish
p.Thr4853Ile

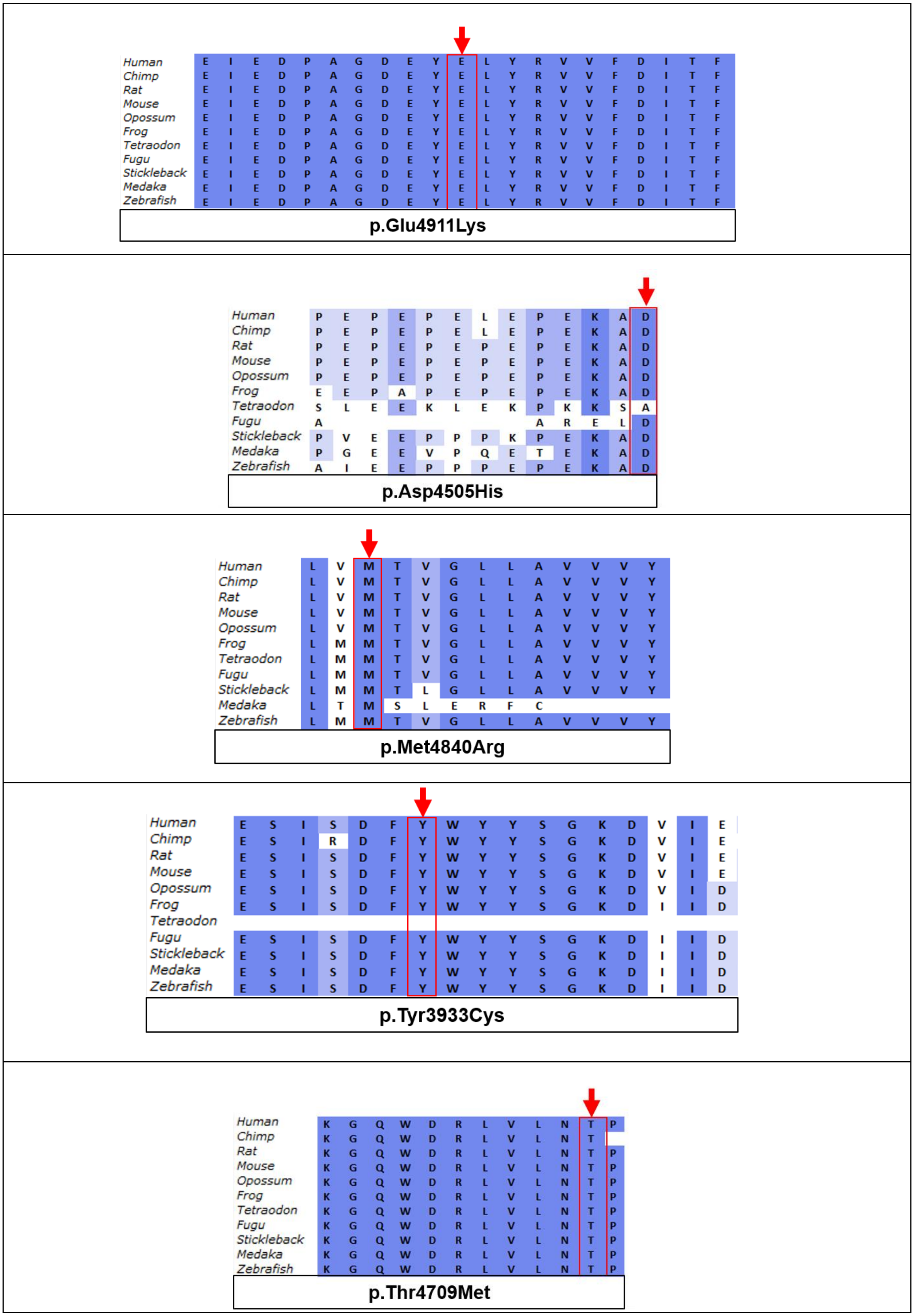
Human
Chimp
Rat
Mouse
Opossum
Frog
Tetraodon
Fugu
Stickleback
Medaka
Zebrafish
p.Glu4911Lys
p.Asp4505His
p.Met4840Arg
p.Tyr3933Cys
p.Thr4709Met

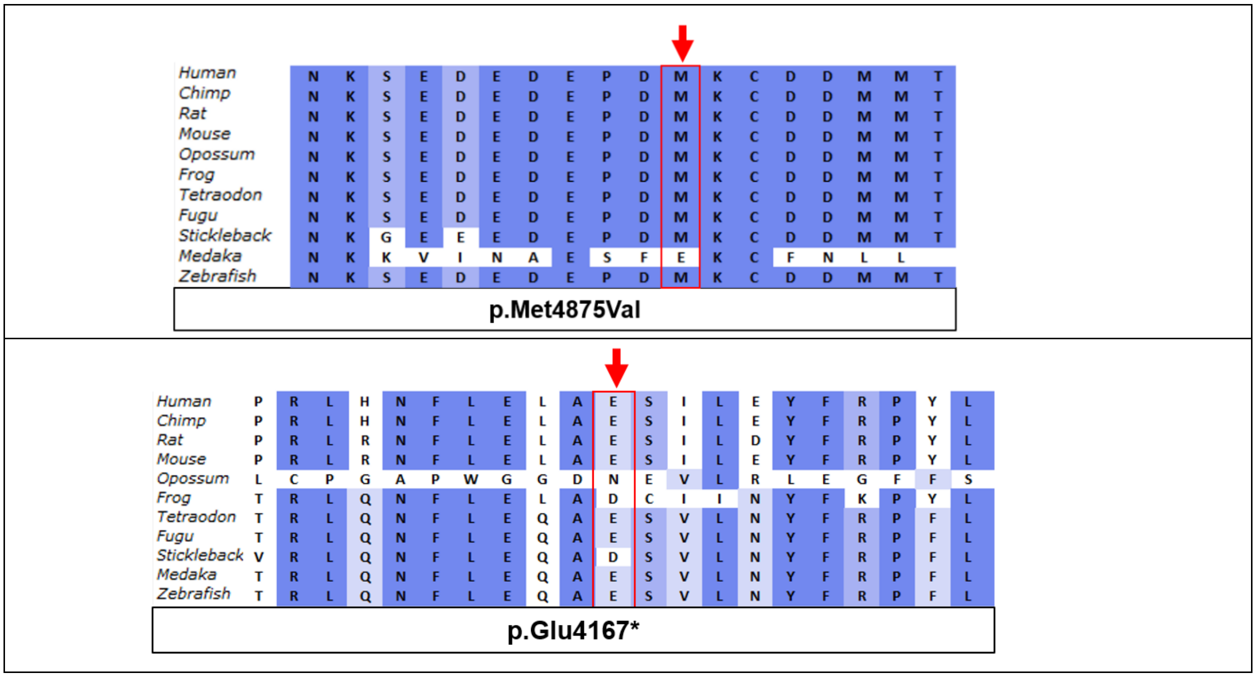


**Figure S2.** Orthologue alignments for each *RYR1* variant affecting the channel and activation core. All affected amino acid residues are highly conserved evolutionarily except p.Gly4444-Gly4450dup.

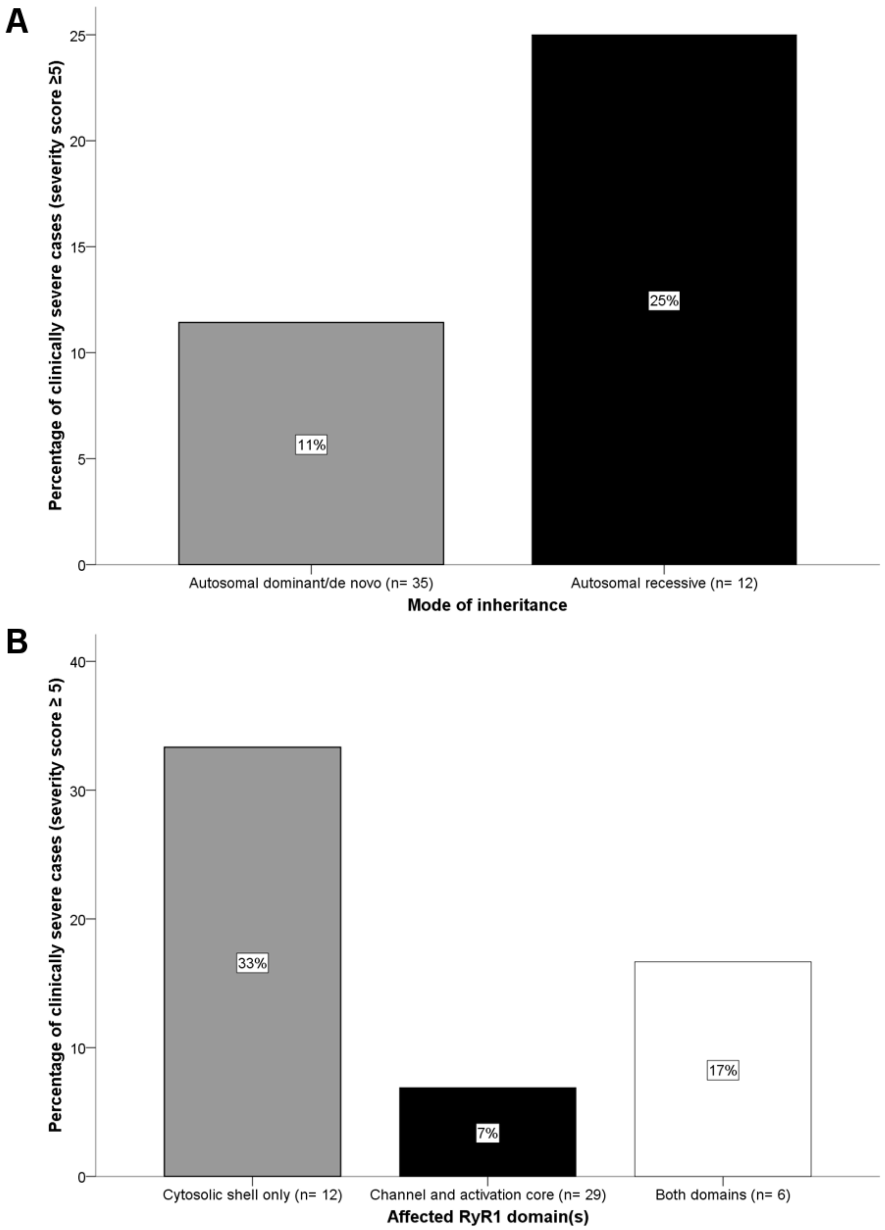


**Figure S3.** Percentage of cases deemed to be clinically severe by mode of inheritance (A) and affected RyR1 domain(s) (B). (A) Although a greater proportion of autosomal dominant/*de novo* cases were deemed clinically severe than recessive cases, this difference was not statistically significant (11% versus 25% respectively, $p = 0.35$). (B) The difference in the proportion of clinically severe cases between those with the RyR1 cytosolic shell affected, compared those with the RyR1 channel and activation core affected bordered statistical significance (33% versus 7% respectively, $p = 0.05$). There were no other statistically significant differences between groups.

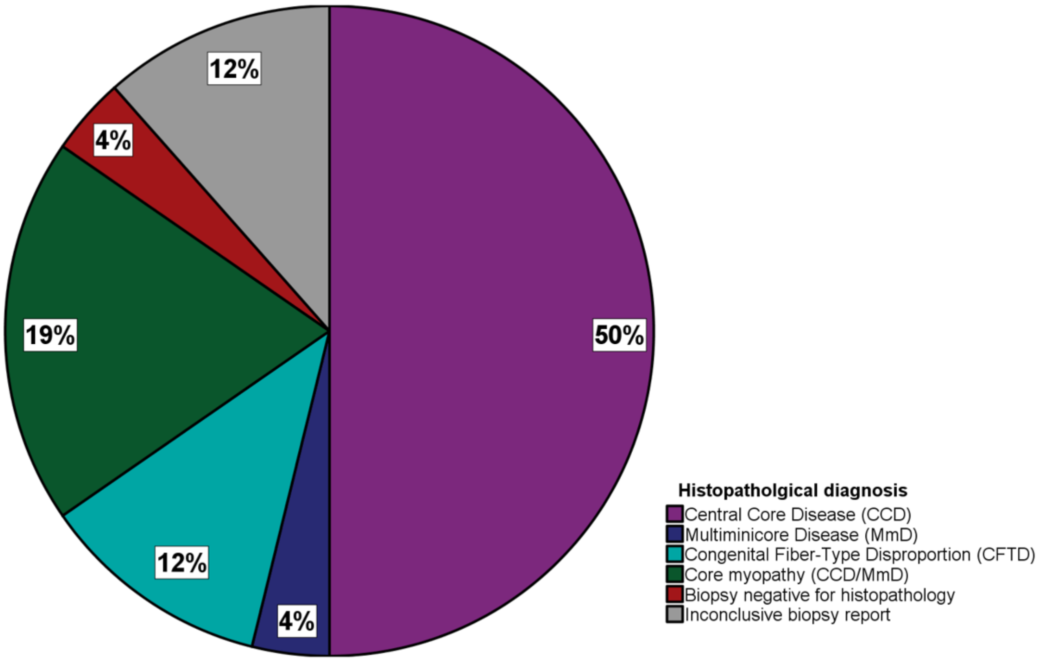


**Figure S4.** Overview of histopathologic diagnoses. Results are representative of participants with skeletal muscle biopsy reports (*n* = 26).

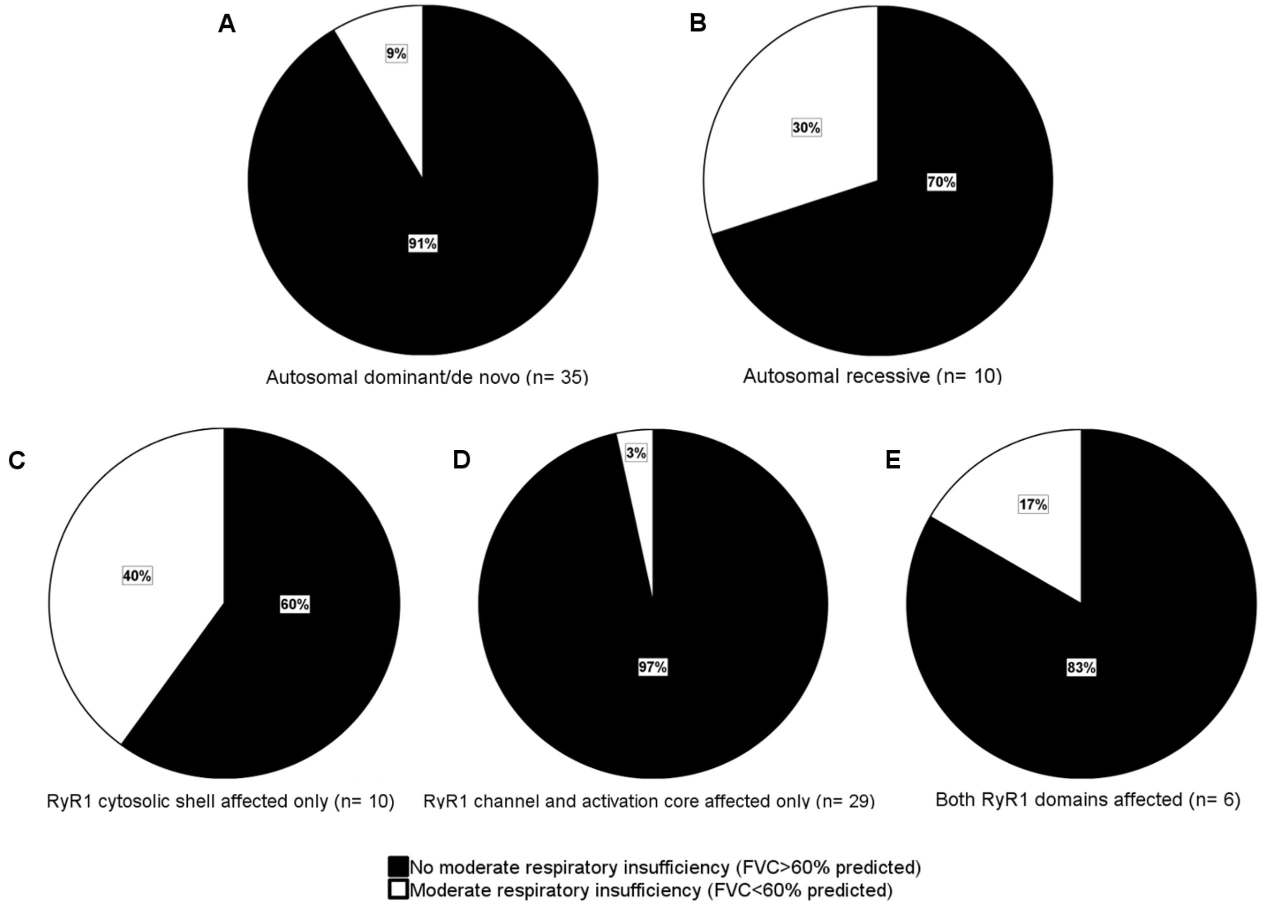


**Figure S5.** Pie charts detailing proportion of individuals with moderate respiratory insufficiency by mode of inheritance (A-B) and affected RyR1 structural domain(s) (C-E). Although a greater proportion of recessive cases exhibited moderate respiratory insufficiency, this difference fell short of statistical significance (30% versus 9% respectively, $p = 0.11$). A greater proportion of participants with the RyR1 cytosolic shell affected only exhibited moderate respiratory insufficiency compared to those with the RyR1 channel and activation core affected only (40% versus 3% respectively, $p = 0.01$). There were no other statistically significant differences between groups.
