# Supplementary material for: Correlation of phenotype with genotype and protein structure in RYR1-related disorders
Source: J Neurol. 2018 Aug 28;265(11):2506–24. doi: 10.1007/s00415-018-9033-2 (PMC6182665; doi:10.1007/s00415-018-9033-2)
Supplement: Supplementary file 3 — Supplementary material 3 (PDF 2562 KB) [file 415_2018_9033_MOESM3_ESM.pdf]

Correlation of phenotype with genotype and protein structure in *RYR1*-related disorders

Supplementary Figures (2)

Joshua J. Todd [1*], Vatsala Sagar [2], Tokunbor A. Lawal [1], Carolyn Allen [1], Muslima S. Razaqyar [1], Monique S. Shelton [1], Irene C. Chrismer [1], Xuemin Zhang [1], Mary M. Cosgrove [1], Anna Kuo [1], Ruhi Vasavada [3], Mina S. Jain [3], Melissa Waite [3], Dinusha Rajapakse [2], Jessica W. Witherspoon [1], Graeme Wistow [2], Katherine G. Meilleur [1].

[1] Neuromuscular Symptoms Unit, National Institute of Nursing Research, National Institutes of Health, Bethesda, MD, United States

[2] Section on Molecular Structure and Functional Genomics, National Eye Institute, National Institutes of Health, Bethesda, MD, United States

[3] Mark O. Hatfield Clinical Research Center, Rehabilitation Medicine Department, National Institutes of Health, Bethesda MD, USA

Figures S6-S39. Positions of human phenotype-related *RYR1* sequence variants based on the latest cryo-EM mammalian (rabbit) RyR1 structure.

Protein structure (PDB: 5TAX) is shown in ribbon diagrams with each tetrameric subunit assigned distinct colors, yellow, blue, cyan, and white. Details of variant residue positions and selected nearby residues are shown in licorice stick models. Main chain and side chain oxygen atoms are shown in red, nitrogen in blue and sulfur in yellow. The affected residue is shown in magenta. Stop-gain, synonymous substitution, and frame-shift variants are not included. The following variants localized to structurally unassigned residues were not mapped: p.Ile1571Val and p.Arg3366His, both Case 43, and p.Asp4505His Cases 39-41. The following figures are organized by variant pathogenicity classification.

Abbreviations: Bsol, bridging solenoid; NTD-B, N-terminal domain B; NTD-A, N-terminal domain A; SPRY1, SP1a/ryanodine receptor domain 1; Nsol, N-terminal solenoid; RY1&2, RYR repeats 1 and 2; SPRY3, SP1a/ryanodine receptor domain 3; Pore, channel pore domain; S6c, cytoplasmic extension of S6; pVSD, pseudo voltage sensor domain; S2S3, helical-bundle domain between S2 and S3.

<table>
<tr><td colspan="2">Classification: Pathogenic</td></tr>
<tr><td>
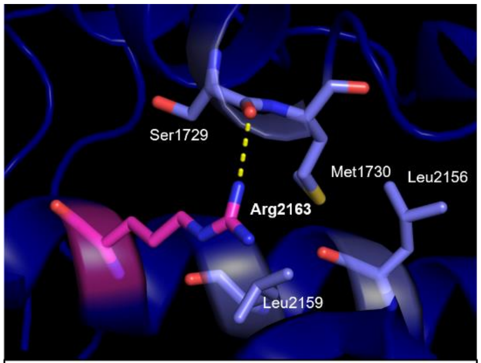

p.Arg2163His (rabbit p.Arg2163)
</td><td>Figure S6. p.Arg2163His (rabbit p.Arg2163). Case 6. Localized to Bsol region. Positively charged Arg2163 (pK_a 12.5) involved in backbone O interaction with p.Ser1728 (rabbit Ser1729. More neutral histidine (pK_a 6.0) variation would alter charge environment. Localized to CS plane of interest. Clinical severity score of 2 and Grantham distance of 29. Letter F in Figure 4.</td></tr>
</table>

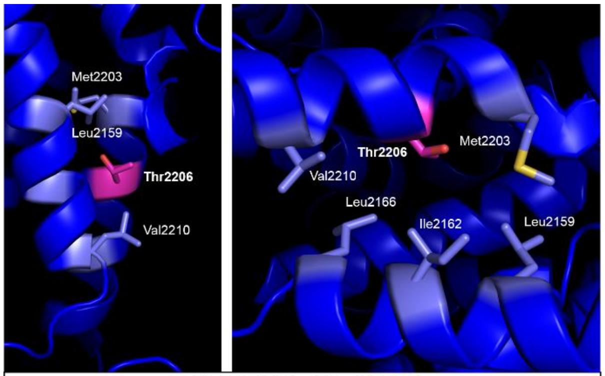

p.Thr2206Met (rabbit p.Thr2206)

Figure S7. p.Thr2206Met (rabbit Thr2206). Case 12. Localized to Bsol region. Thr is solvent exposed. Met is non-polar and has a larger side chain which may cause neighboring helices to be pushed apart. Severity score of 4 and Grantham distance of 81. Localized to CS plane of interest. Letter G in Figure 4.

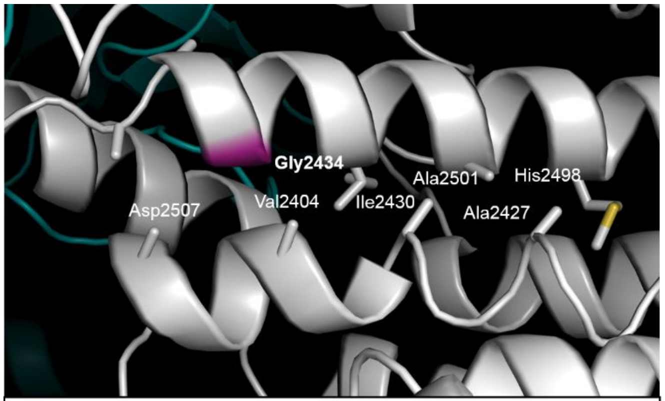

p.Gly2434Arg (rabbit p.Gly2434)

Figure S8. p.Gly2434Arg (rabbit p.Gly2434). Case 47. Localized to Bsol region. Gly2434 occupies a hydrophobic environment between helices. Substitution with bulky, cationic Arg may disrupt α helix packing. Clinical severity score of 4 when co-expressed with p.Met4875Val (rabbit 4874) (variant of uncertain significance) and a Grantham distance of 125. Localized to CS plane of interest. Letter J in Figure 4.

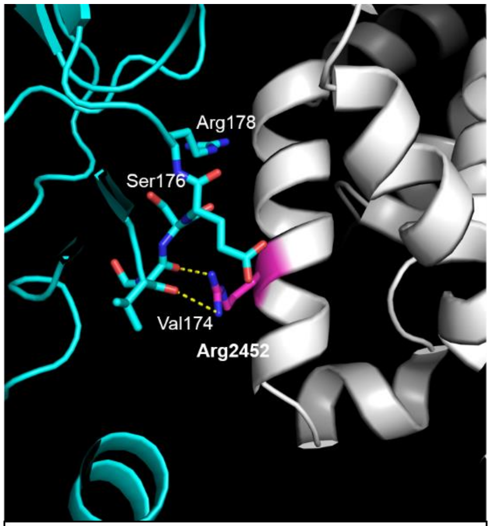

p.Arg2452Trp (rabbit p.Arg2452)

Figure S9. p.Arg2452Trp. (rabbit p.Arg2452) Cases 1 and 2. Arg2452 in the Bsol region makes intermolecular interaction with NTD-A. NH1 and NH2 atoms hydrogen bond with backbone carbonyls of p,Val173 (rabbitVal174) and p.Ser175(rabbit Ser176). A tryptophan substitution will reduce the H-bond donor potential, likely causing local rearrangement Clinical severity score of 4. Localized to CS plane of interest. Letter L in Figure 4.

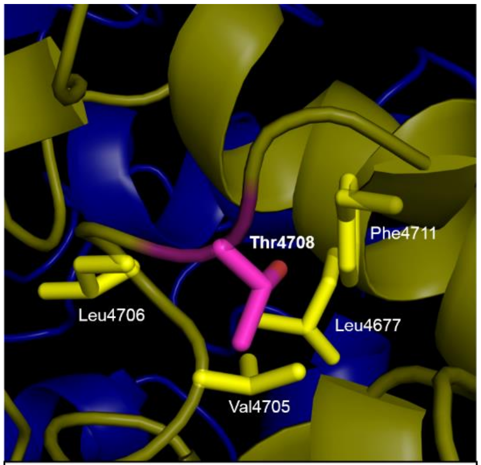<br>p.Thr4709Met (rabbit p.Thr4708)	**Figure S10. p.Thr4709Met (rabbit p.Thr4708).** Case 44. Localized to pVSD region. Thr4708 has a small side chain relative to Met which may be too large to conform to the wild-type structure. Clinical severity score of 4, when co-expressed with p.Arg2241*, and a Grantham distance of 81. Loop between S2 and S3, critical to RyR1opening [6]. Letter Q in Figure 4.
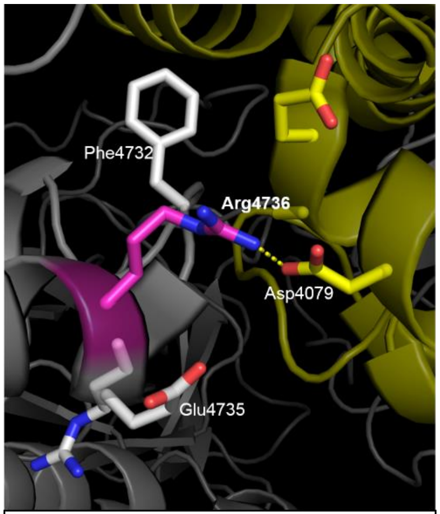<br>p.Arg4737Gln (rabbit p.Arg4736)	**Figure S11. p.Arg4737Gln (rabbit p.Arg4736).** Case 34. Localized to pVSD region, specifically within the S2S3 helical bundle. In the closed form, Arg4736 forms an inter-subunit ion pair with p.Asp4078 (rabbit Asp4079). Substitution with the shorter, neutral glutamine would modify the charge interaction. Clinical severity score of 2 when co-expressed with both of the following: p.Met4022Thrfs*4 and c.6797-9C>T (intronic). Grantham distance of43. Critical region for RyR1 opening [6]. Letter P in Figure 4.
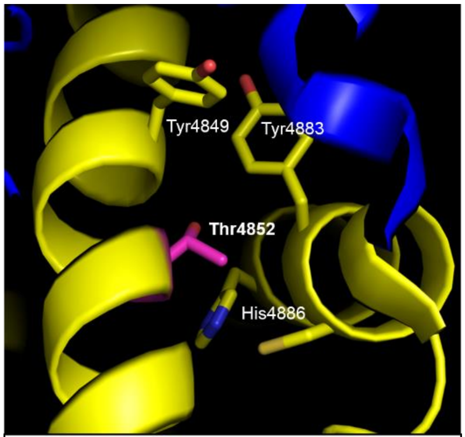<br>p.Thr4853Ile (rabbit p.Thr4852)	**Figure S12. p.Thr4853Ile (rabbit p.Thr4852).** Case 37. Localized to pore region. Thr4852 in polar intramolecular interaction. Substitution with nonpolar, and slightly larger, isoleucine will change polarity and packing of environment. Clinical severity of 4 and Grantham distance of 89. Letter X in Figure 4.

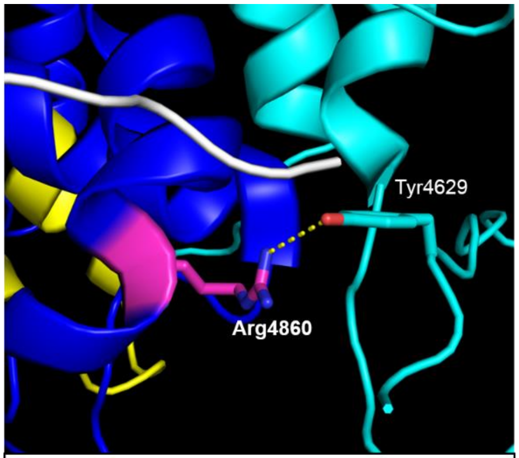

p.Arg4861His (rabbit p.Arg4860)

Figure S13. p.Arg4861His (rabbit p.Arg4860). Cases 23, 24, 25, 26, 27, 32. Localized to pVSD region. Arg4860 (pK_a 12.5) makes inter-subunit polar contact with hydroxyl group of p.4630 (rabbit Tyr4629). It is at the tip of a negatively charged loop. Substitution with more neutral histidine (pK_a 6) would alter electrostatic properties of the region. May affect luminal triadin binding and retention of RyR-CSQ proximity and ability for rapid Ca^{2+} release [3, 4]. Grantham distance of 29 and average clinical severity score of 3 when expressed alone (Cases 23-27). Clinical severity score of 2 when co-expressed with p.Gly4444-Gly4450dup (Case 32). Letter Z in Figure 4.

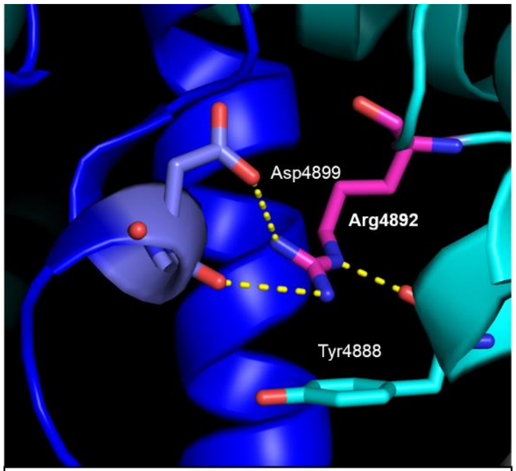

p.Arg4893Gln (rabbit p.Arg4892)

Figure S14. p.Arg4893Gln (rabbit p.Arg4892). Cases 28 and 29. Localized to pore region, adjacent to the selectivity filter. Rabbit Arg4892 forms an inter-subunit ion pair with p.Asp4900 (rabbit Asp4899) and hydrogen bonds with backbone carbonyls of p.Tyr4889 (rabbit Tyr4888) and p.Asp4900 (rabbit Asp4899). Substitution with neutral glutamine would weaken or eliminate these interactions. Clinical severity of 4 and a Grantham distance of 43. Luminal triadin binding and may affect retention of RyR-CSQ proximity and ability for rapid Ca^{2+} release [3, 4]. Letter W in Figure 4.

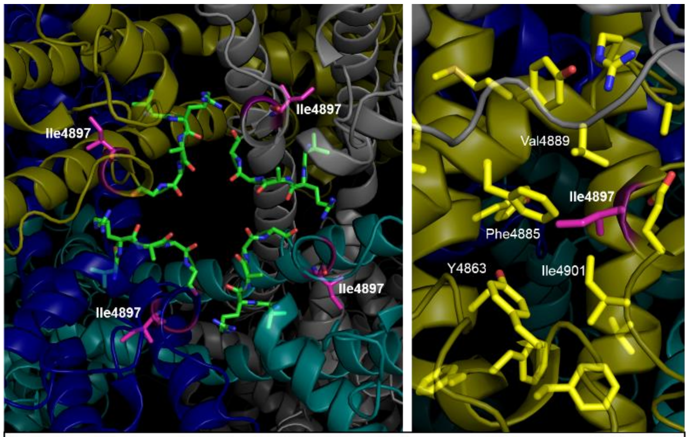

p.Ile4898Thr (rabbit p.Ile4897)

Figure S15. p.Ile4898Thr (rabbit p.Ile4897). Cases 15 and 16. Localized to pore region. Residues of the selectivity filter in the ion channel pore are shown with carbon atoms in green. Hydrophobic residues, making intramolecular contacts with Ile4897 are shown in yellow. Ile4897 contributes to hydrophobic packing around the base of the pore. Substitution with polar Thr would likely disrupt this organization. Clinical severity score of 2 and Grantham distance of 89. Letter W in Figure 4.

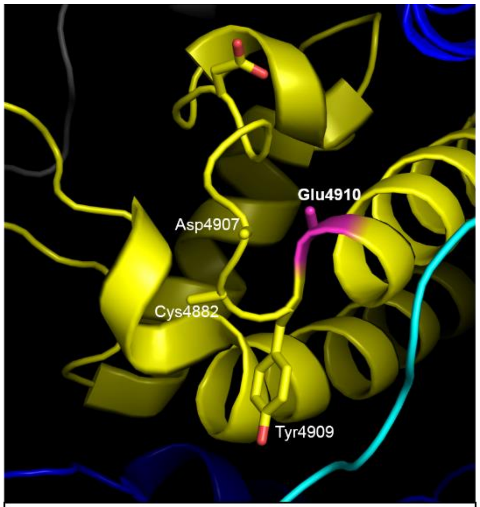

p.Glu4911Lys (rabbit p.Glu4910)

Figure S16. p.Glu4911Lys (rabbit p.Glu4910). Cases 38 and 43. Localized to periphery of pore region. Negatively charged Glu4910 in polar environment. Substitution with positively charged lysine would alter charge environment. Grantham distance of 56. Clinical severity score of 2 when expressed alone (Case 38). Clinical severity score of 5 when co-expressed with all of the following p.Ile1571Val, p.Arg3366His, and p.Tyr3933Cys (Case 43). Letter Y in Figure 4.

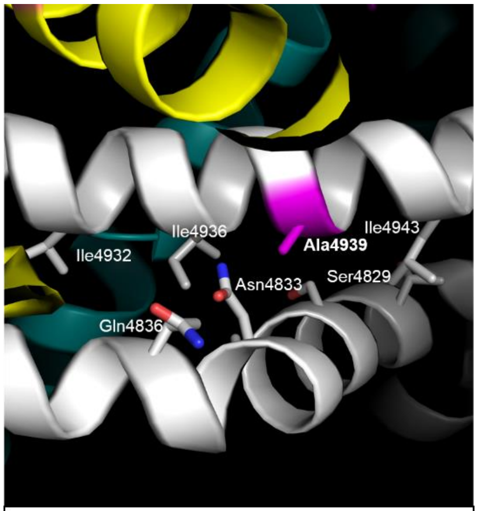

p.Ala4940Thr (rabbit p.Ala4939)

Figure S17. Ala4940Thr (rabbit p.Ala4939). Cases 17, 18, 19, and 45. Localized to S6c cytoplasmic extension in pore region, implicated in channel gating. Alanine lines the side of a hydrophobic helix and its small size allows for close packing between S6 and the adjacent helix, S5. Grantham distance of 58. Clinical severity score was 2 when expressed alone and also when co-expressed with p.Arg2224His. This variation may also affect ATP-mediated channel activation due to its proximity to the binding interface [1]. Letter U in Figure 4.

Classification: Variant of uncertain significance

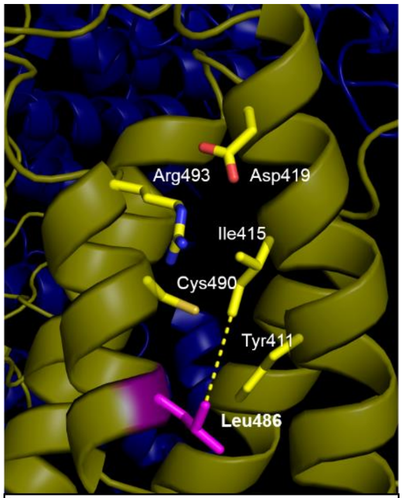

p.Met485Val (rabbit p.Leu486)

Figure S18. p.Met485Val (rabbit p.Leu486). Case 5. Localized to surface of Nsol region. Conservative non-polar variant; smaller side chain. Grantham distance of 21. Clinical severity score of 4 when co-expressed with all of the following: p.Arg2241*, p.Arg109Trp, and p.Asp708Asn. Localized to CS plane of interest. Single nucleotide variant dbSNP# 147723844 [7]. Letter E in Figure 4.

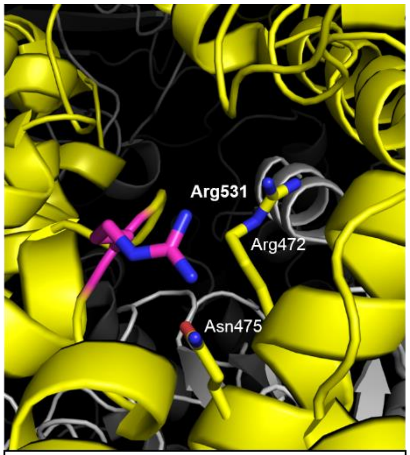 p.Arg530His (rabbit p.Arg531)	**Figure S19. p.Arg530His (rabbit p.Arg531).** Case 7. Localized to Nsol region. Positively charged Arg (pK_a 12.5) exposed to aqueous cavity. More neutral His (pK_a 6) variation would alter charge environment. Clinical severity score of 2 and localized to CS plane of interest. Letter D in Figure 4.
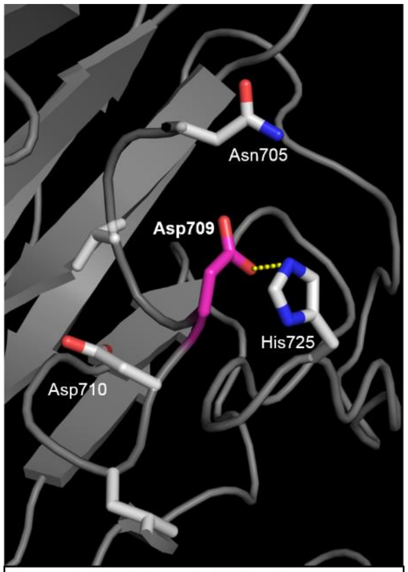 p.Asp708Asn (rabbit p.Asp709)	**Figure S20. p.Asp708Asn (rabbit p.Asp709).** Case 5. Localized to SPRY1 region. Conservative variant swaps charged side chain for neutral; Grantham distance of 23. Clinical severity score of 4 when co-expressed with all of the following: p.Arg2241*, p.Arg109Trp, p.Asp708Asn, p.Met485Val. In rabbit, Asp709 makes polar interactions with p.Arg682 (rabbit Arg683) and p.His724 (rabbit His725), most of which would be preserved on substitution by Asn. This is an FKBP interaction site and may affect channel stabilization. Letter B in Figure 4.
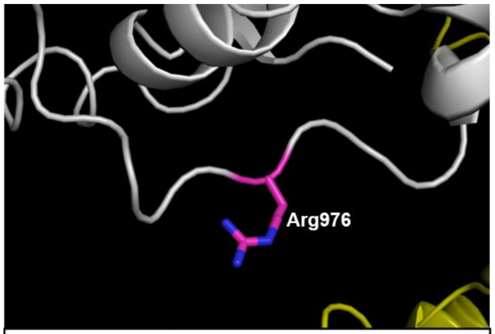 p.Arg975Trp (rabbit p.Arg976)	**Figure S21. p.Arg975Trp (rabbit p.Arg976).** Cases 8 and 9. Localized to RY1&2 region (flexible tandem repeat). Charged Arg is exposed to solvent. The more aromatic tryptophan may avoid water, possibly causing local rearrangements. Severity score of 2, despite Grantham score of 101, suggests residue may not

<table>
<tr><td></td><td>be crucial for function. Site of inter-subunit interaction. Letter A in Figure 4.</td></tr>
<tr><td>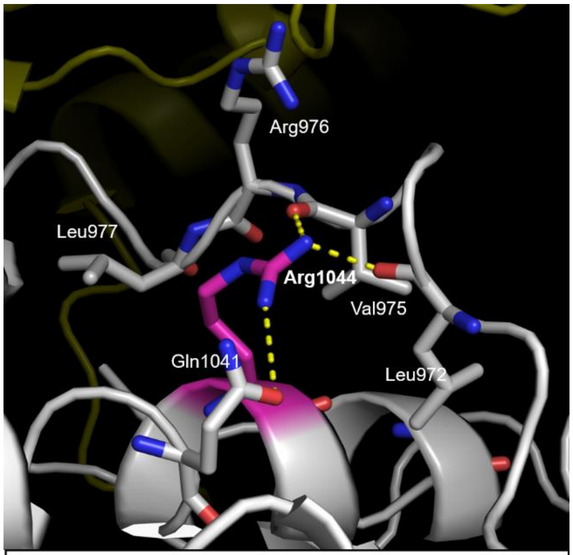

p.Arg1043Cys (rabbit p.Arg1044)</td><td>Figure S22. p.Arg1043Cys (rabbit p.Arg1044). Case 7. Localized to RY1&2 region (flexible tandem repeat). Large, charged Arg interacting with backbone of p.Val974 (rabbit Val975) and p. Leu 971 (rabbit Leu972), as well as p. Gln 1040 (rabbit Gln1041), changed for small, non-polar Cys. Severity score of 2, when co-expressed with both Arg530His and Arg2336His. The low clinical severity score, despite Grantham distance of 180, suggests that this amino acid may not be crucial for function. Site of inter-subunit interaction. Letter A in Figure 4.</td></tr>
<tr><td>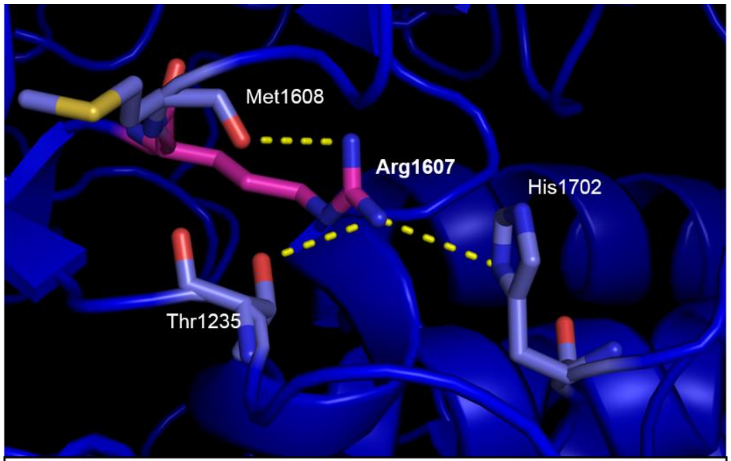

p.Arg1606His (rabbit p.Arg1607)</td><td>Figure S23. p.Arg1606His (rabbit p.Arg1607). Case 47. In rabbit, charged Arg1607 (pK_a 12.5) contacts His1701 (rabbit His1702), Thr1234 (rabbit Thr1235), and backbone carbonyl of Met1607 (rabbit Met1608). More neutral histidine variation (pK_a 6) may change these interactions. Site of inter-RyR1 interaction. Clinical severity score of 3 when co-expressed with both p.Gly1165Gly and p.Glu4167*. Letter C in Figure 4.</td></tr>
</table>

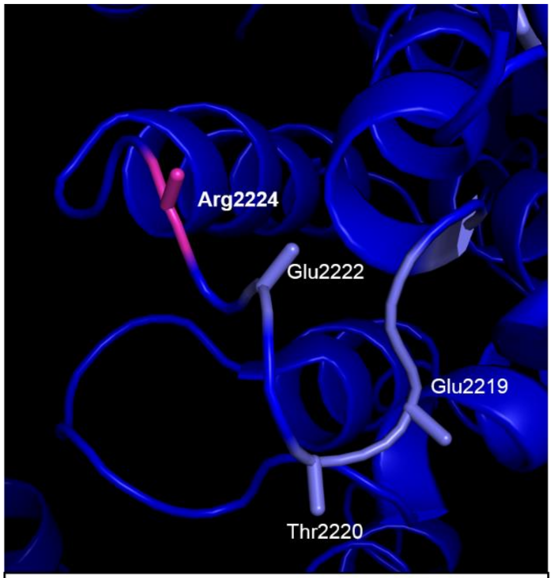

p.Arg2224His (rabbit p.Arg2224)

Figure S24. p.Arg2224His (rabbit p.Arg2224). Case 45. Localized to Bsol region. Positively charged Arg2224 (pK_a 12.5) facing aqueous cavity. More neutral histidine variation (pK_a 6) would alter charge environment. Clinical severity score of 2, when co-expressed with p.Ala4940Thr, and a Grantham distance of 29. Localized to CS plane of interest. Letter H in Figure 4.

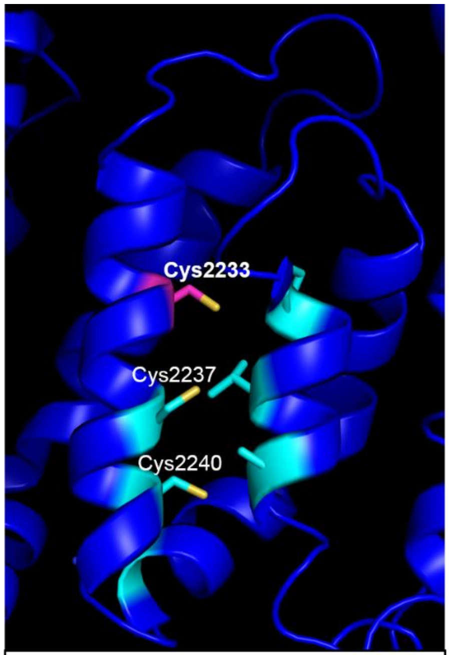

p.Cys2233Arg (rabbit p.Cys2233)

Figure S25. p.Cys2233Arg (rabbit p.Cys2233). Case 4. Localized to Bsol region and lines α-helix with adjacent cysteines. Cys2233 participates in hydrophobic interactions with neighboring helix. Charged, bulky Arg variation likely to disrupt α helix packing. Clinical severity score of 4 and Grantham distance of 180. Letter I in Figure 4.

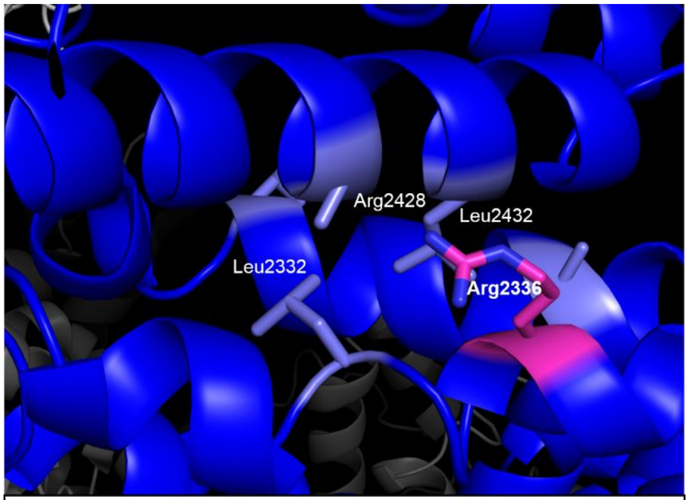

p.Arg2336His (rabbit p.Arg2336)

Figure S26. p.Arg2336His (rabbit p.Arg2336). Case 7. Localized to Bsol region. Positively charged Arg2336 (pK_a 12.5) facing aqueous solution. More neutral histidine variation (pK_a 6) would alter charge environment. Possibly involved in interaction between Csol and Nsol of same monomer and NTD of adjacent monomer. Grantham distance of 29. Clinical severity score of 2 when co-expressed with both of the following: p.Arg530His and p.Arg1043Cys. Localized to CS plane of interest. Letter J in Figure 4.

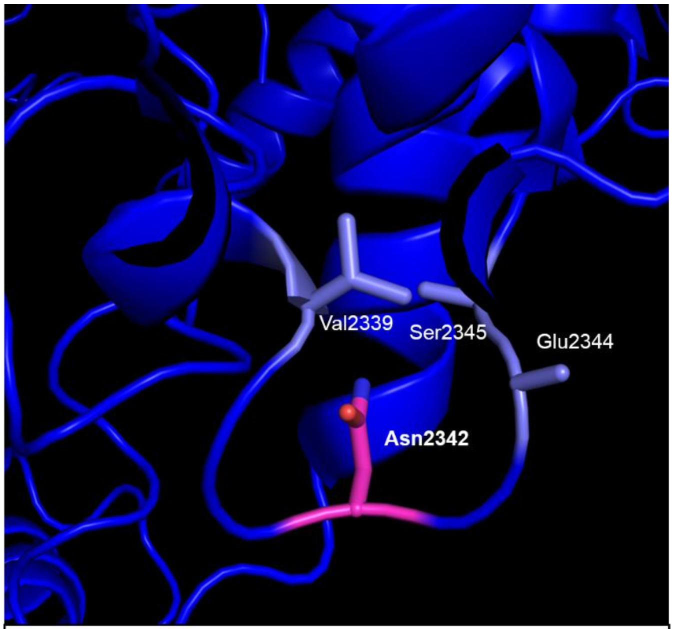

p.Asn2342Ser (rabbit p.Asn2342)

Figure S27. p.Asn2342Ser (rabbit p.Asn2342). Case 42. Localized to Bsol region. Substitution of Asp with Ser retains polarity with a smaller side chain. Site of inter-protomer contact with NTD-A [1]. redundant Grantham distance of 46. Clinical severity score of zero is consistent with this being a conservative variant. Localized to CS plane of interest. Letter J in Figure 4. Variant was co-expressed with p.Met4840Arg.

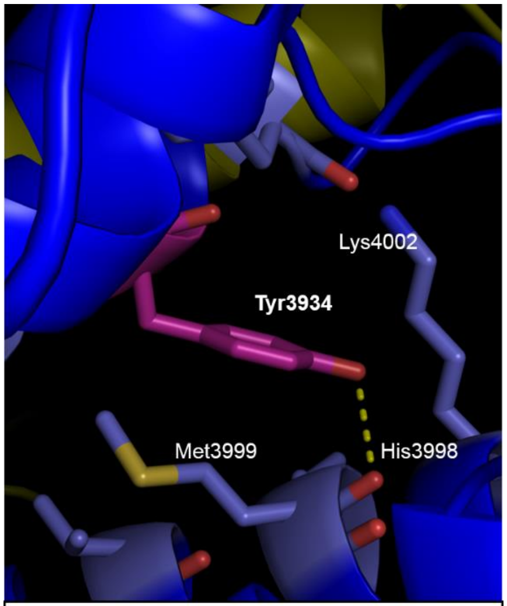<br>p.Tyr3933Cys (rabbit p.Tyr3934)	**Figure S28. p.Tyr3933Cys (rabbit Tyr3934).** (Case 43). Localized to Csol region. Tyr3934 is facing aqueous solution and may be involved with intramolecular contact with backbone atom of p.His3997 (rabbit His3998). Substitution with Cys would leave this residue prone to oxidation. Possibly involved in interaction between Csol and Bsol of same monomer and NTD of adjacent monomer. Close to putative Ca^{2+} binding site [1]. Localized to CS plane of interest. Letter M in Figure 4.
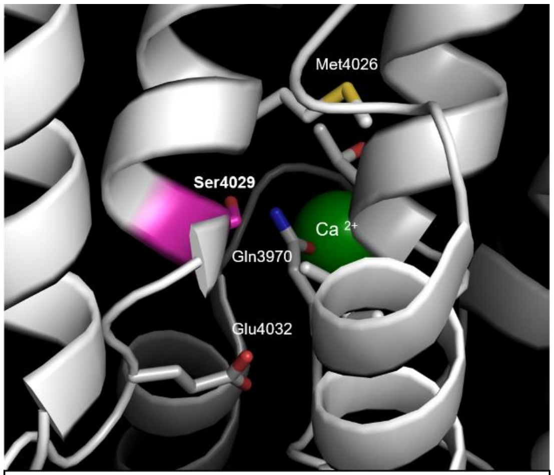<br>p.Ser4028Leu (rabbit p.Ser4029)	**Figure S29. p.Ser4028Leu (rabbit p.Ser4029).** Case 35. Localized to Csol region. Ser4029 involved in polar intramolecular interaction between helices. Substitution with nonpolar Leu may disrupt this interaction. Close to putative Ca^{2+} binding site [1]. Clinical severity score of 2 and Grantham distance of 145 suggests amino acid may not be crucial for function. Letter N in Figure 4.

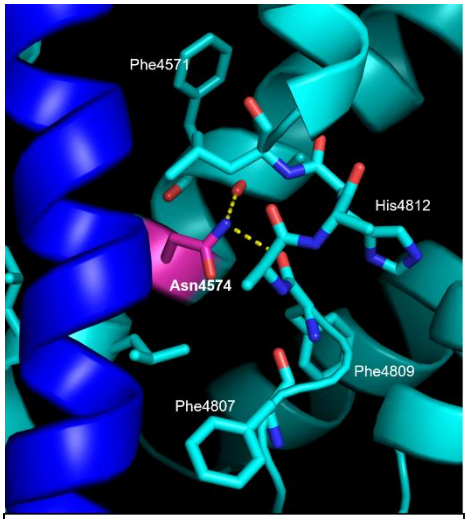

p.Asn4575Thr (rabbit p.Asn4574)

Figure S30. p.Asn4575Thr (rabbit p.Asn4574). Case 55. Localized to pVSD region. Rabbit Asn4574 is a polar residue surrounded by aromatics p.Phe4572 (rabbit Phe4571), and hydrophobic groups p.Leu 4572 (rabbit Leu4577) and p. Leu4808 (rabbit Leu4813). Asn 4574 hydrogen bonds to backbone of p.Phe4803 (Phe4809). Substitution with threonine is conservative. Clinical severity of 4 and Grantham distance of 21 suggests amino acid may be important for function. Letter S in Figure 4.

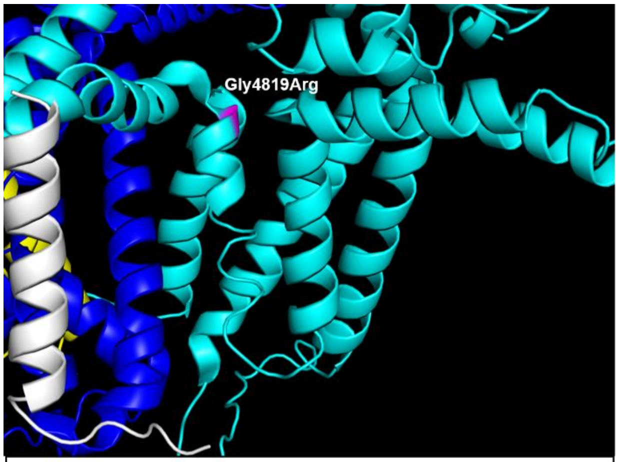

p.Gly4820Arg (rabbit p.Gly4819)

Figure S31. p.Gly4820Arg (rabbit p. Gly4819). Cases 20, 21, and 22. Localized to pore region at the junction between the SR membrane and cytosol. Gly4820 (rabbit Gly4819) allows turn of the α helix. Non-conservative substitution with Arg may disrupt geometry of the turn. A clinical severity score of 4 indicates the potential importance of this structural feature. Letter R in Figure 4.

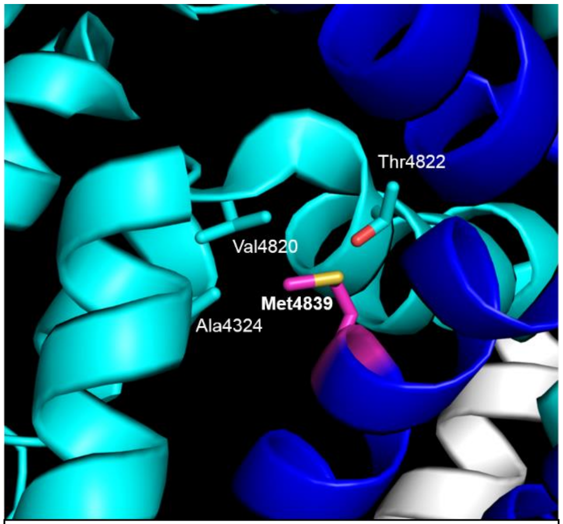

p.Met4840Arg (rabbit p.Met4839)

Figure S32. p.Met4840Arg (rabbit p.Met4839). Case 42. Localized to pore region. Met4839 is involved in hydrophobic intermolecular interaction at sharp turn in adjacent helix. Substitution with cationic Arg is expected to alter this channel-stabilizing inter-domain interaction [2]. Clinical severity score of 0 when co-expressed with p.Asn2342Ser suggesting this is not critical for function. Grantham distance of 91. Letter V in Figure 4.

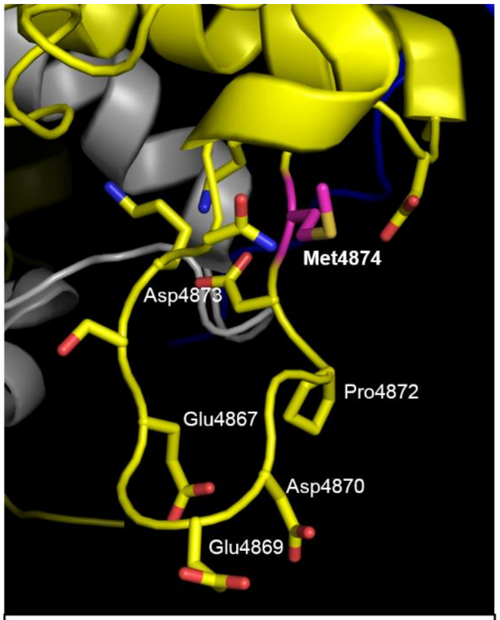

p.Met4875Val (rabbit p.Met4874)

Figure S33. p.Met4875Val (rabbit p.Met4874). Case 46. Localized to pore region. In rabbit, Met4875 is sandwiched between p.Asn4859 (rabbit Asn4864) and p.Asp4872 (rabbit Asp4877), interactions that could require the polarizability of the S atom in the Met sidechain, which would be lost in the Val substitution. Four of these loops frame the exit of the pore. This is a luminal site for triadin binding and may affect retention of RyR-CSQ proximity and ability for rapid Ca^{2+} release [3, 4]. Grantham distance of 21 and clinical severity score of 4 when co-expressed with p.Gly2434Arg. Letter Y in Figure 4.

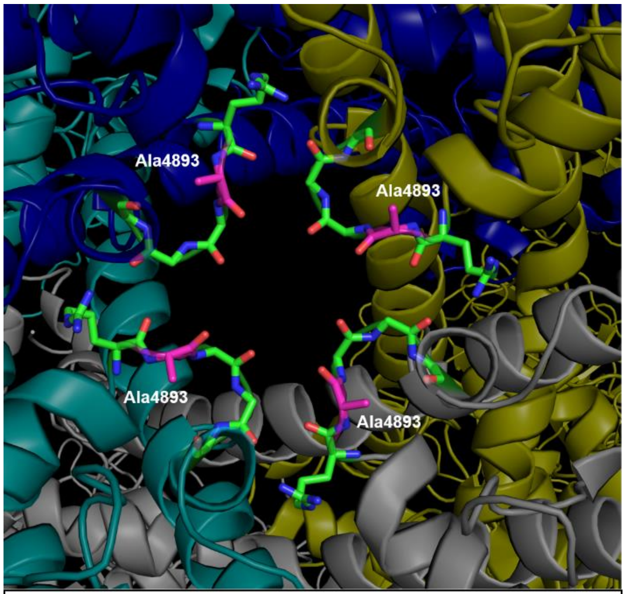

p.Ala4894Asp (rabbit p.Ala4893)

Figure S34. p.Ala4894Asp (rabbit p.Ala4893). Cases 30 and 31. Localized to pore region. Component of the pore itself. Substitution to negatively charged aspartic acid likely affects the conductance or release of Ca^{2+} ions. Clinical severity score of 4 and Grantham distance of 126.Luminal triadin binding [4]. Letter W in Figure 4.

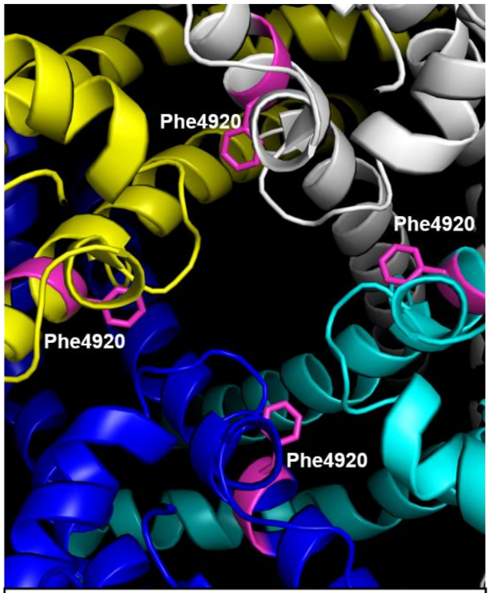

p.Phe4921Leu (rabbit p.Phe4920)

Figure S35. p.Phe4921Leu (rabbit p.Phe4920). Case 14. Localized to pore region. Phe contributes to the packing between pore-lining helices. The smaller side chain of leucine would destabilize the helix packing. Residue is also involved in binding ryanodine [5]. Grantham distance of 22 and clinical severity score of 2. Letter W in Figure 4.

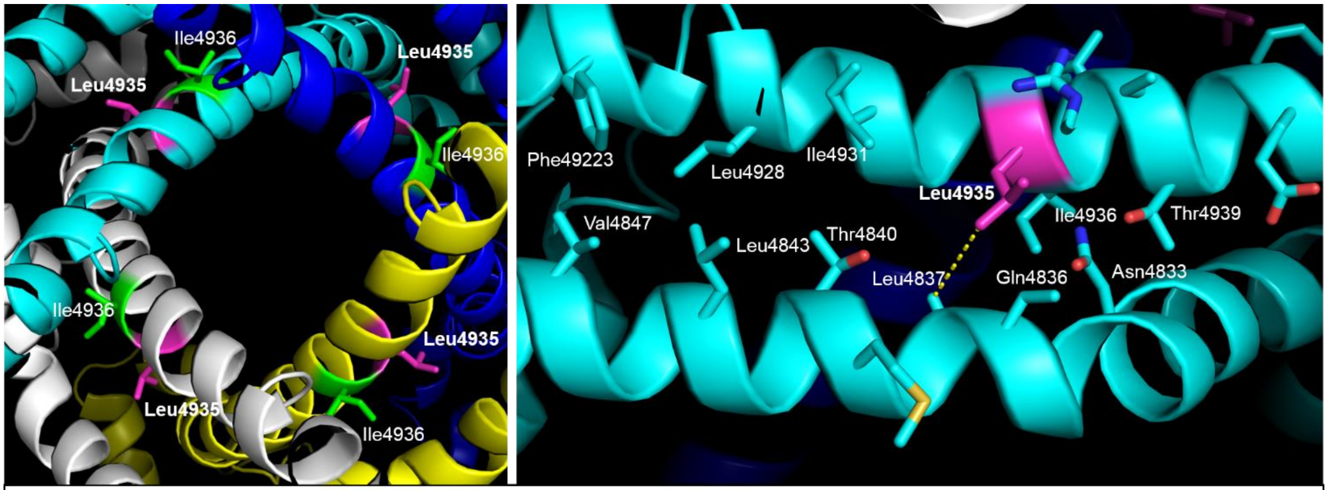

p.Leu4936Arg (rabbit p.Leu4935)

Figure S36. p.Leu4936Arg (rabbit p.Leu4935). Case 33. Localized to helix S5 in pore region at the junction of SR membrane and cytosol. Immediately adjacent to Ile4936, a critical residue for channel gating. Rabbit Leu4935 is involved in hydrophobic interaction between adjacent pore helices S5 and S6. Substitution with large, cationic arginine may disrupt this interaction. Clinical severity score of 4 and a Grantham distance of 102. Letter U in Figure 4.

Classification: Likely pathogenic

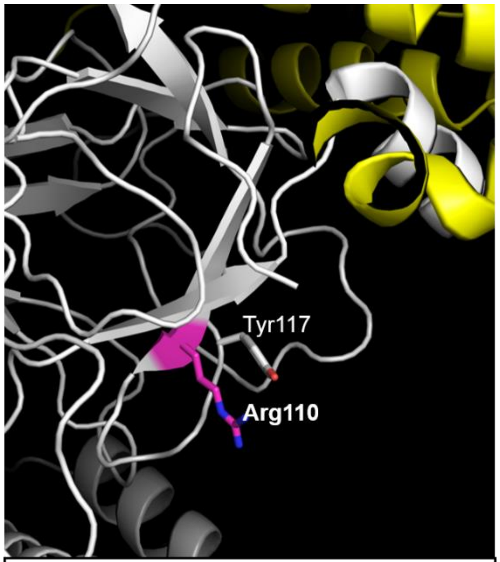

p.Arg109Trp (rabbit p.Arg110)

Figure S37. p.Arg109Trp (rabbit p.Arg110). Case 5. Localized to NTD-A region. Charged Arg is exposed to solvent. The more hydrophobic tryptophan variation prefers to avoid water. Grantham distance of101. Clinical severity score of 4 when co-expressed with all of the following: Arg2241*, Asp708Asn, and Met485Val. Possibly involved in interaction between NTD and Nsol of the same monomer and Bsol of the adjacent monomer. Localized to CS plane of interest. Letter K in Figure 4.

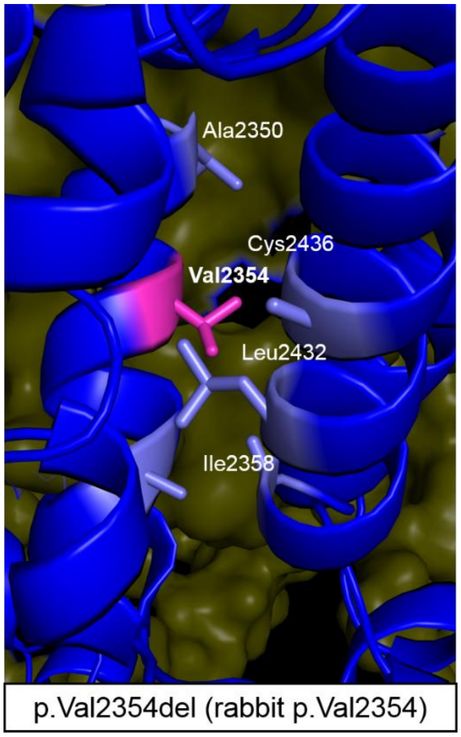	**Figure S38. p.Val2354del (rabbit p.Val2354).** Case 11. Localized to Bsol region. Deletion of Val2354 causes a single residue frame-shift affecting a short α helix, by replacing Val2354 with Arg2355. Val2354 makes a hydrophobic interaction between adjacent helices. Cationic Arg is likely to disrupt this interaction. Localized to CS plane of interest. of adjacent monomer. Clinical severity score of 7 when co-expressed with p.Trp1495*. Letter J in Figure 4.
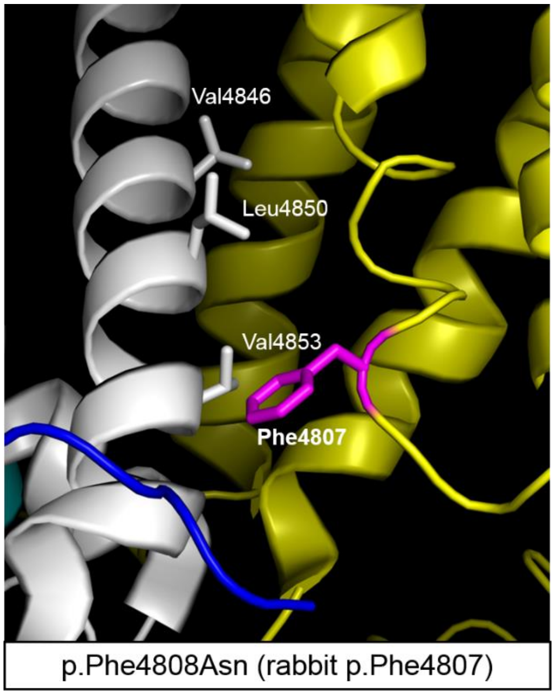	**Figure S39. p.Phe4808Asn (rabbit p.Phe4807).** Case 36. Localized to pVSD. Phe4807 involved in hydrophobic intermolecular interaction between loop region and adjacent helix. Substitution with polar Asn would alter this interaction. Despite a Grantham distance of 158, the clinical severity score of 2 suggests residue may not be crucial for function. Linked to S2S3 and critical for RyR1 opening [6]. Letter T in Figure 4.

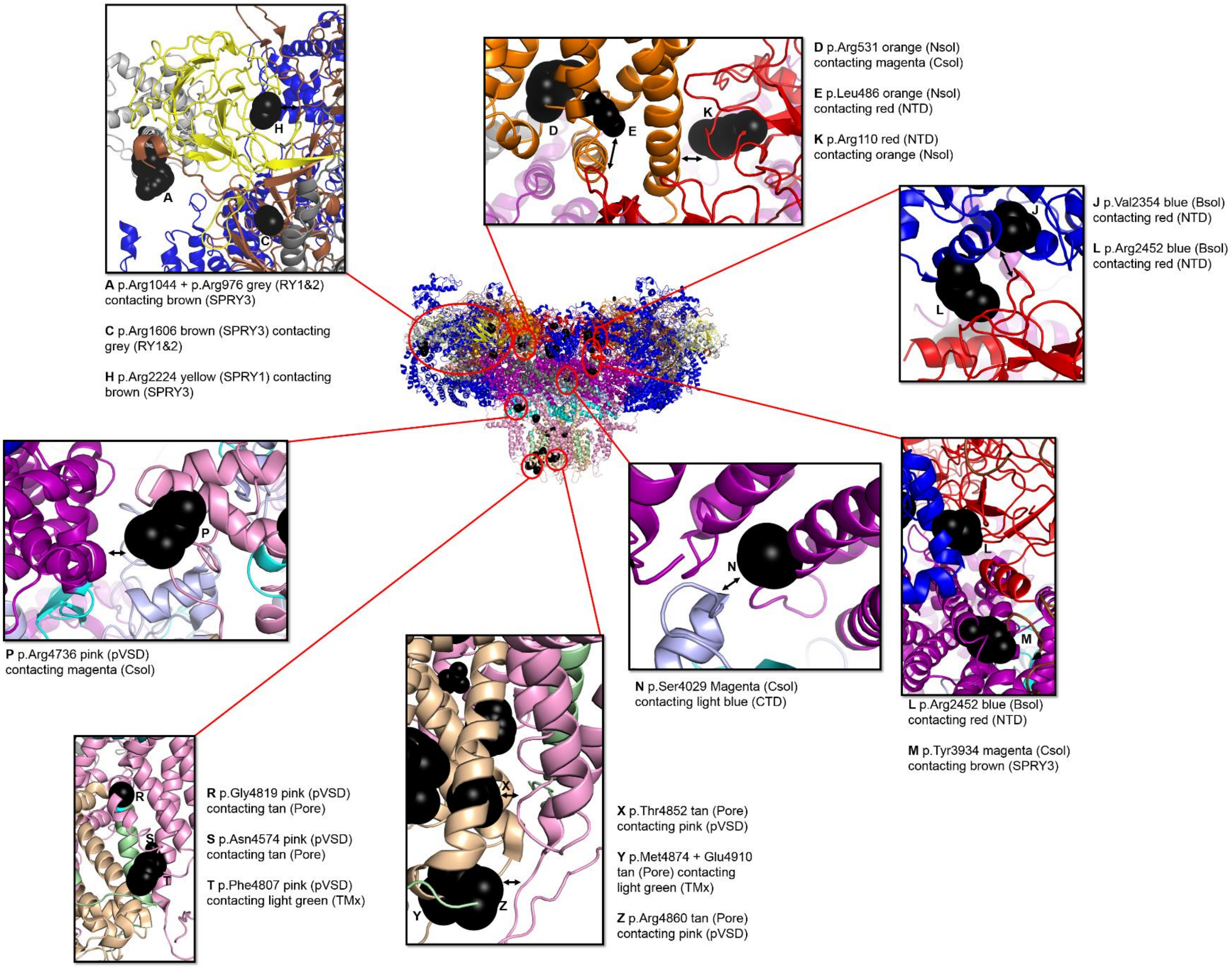

Figure S40. Affected residues that are at the interface of distinct RyR1 regions (rabbit residue numbering).

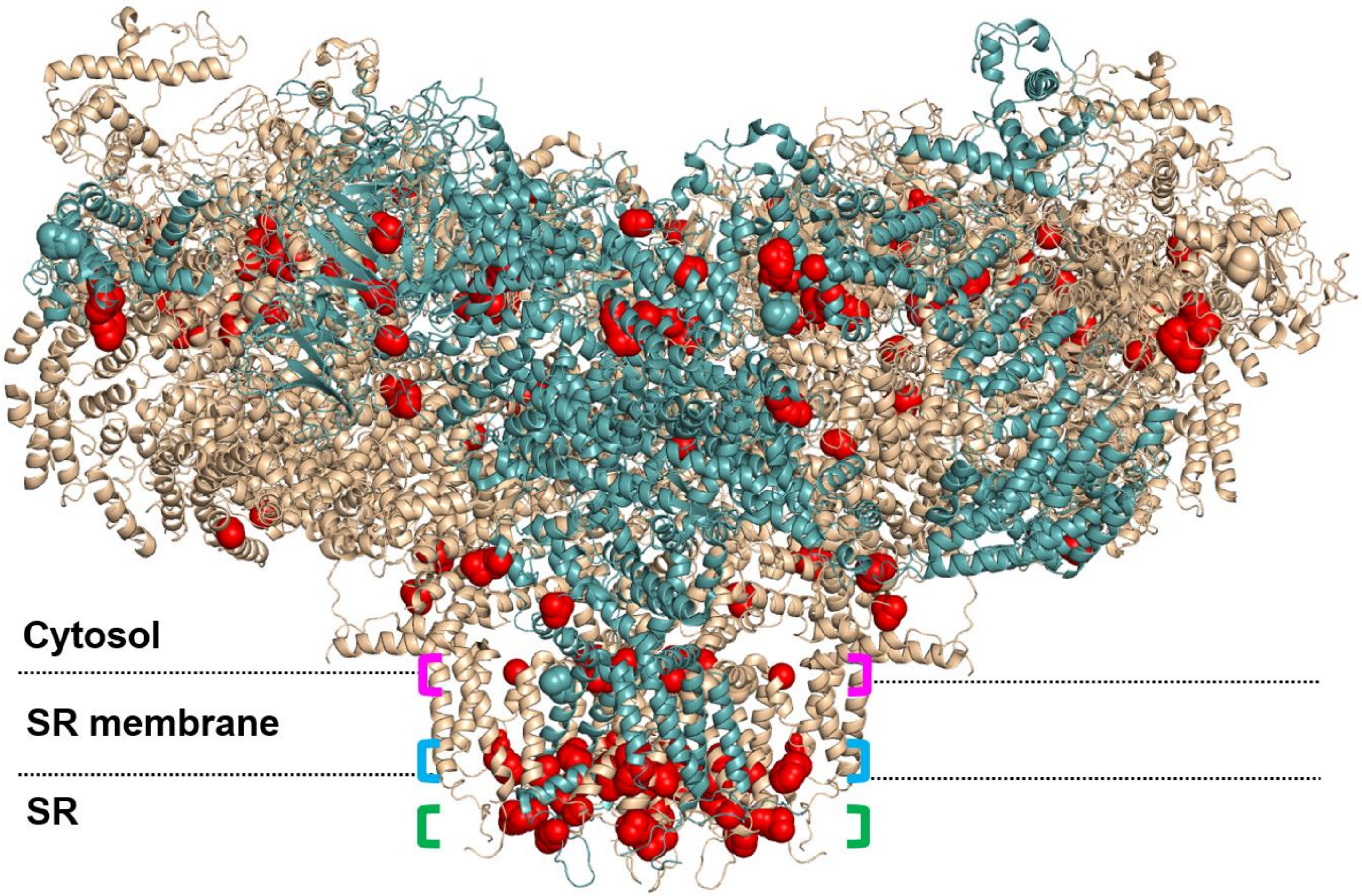

Figure S41. Structural planes of interest in the RyR1 channel and activation core domain. Plane 1 (green brackets) joins luminal loops with the pore region; plane 2 (blue brackets) is localized to the Ca^{2+} entry pore; plane 3 (pink brackets) is where the SR membrane transitions into the cytosol.

References

1 des Georges A, Clarke OB, Zalk R, Yuan Q, Condon KJ, Grassucci RA, Hendrickson WA, Marks AR, Frank J (2016) Structural basis for gating and activation of RyR1. Cell 167: 145-157.e117 Doi 10.1016/j.cell.2016.08.075

2 Hwang JH, Zorzato F, Clarke NF, Treves S (2012) Mapping domains and mutations on the skeletal muscle ryanodine receptor channel. Trends in molecular medicine 18: 644-657 Doi 10.1016/j.molmed.2012.09.006

3 Lee CS, Hanna AD, Wang H, Dagnino-Acosta A, Joshi AD, Knoblauch M, Xia Y, Georgiou DK, Xu J, Long Cet al (2017) A chemical chaperone improves muscle function in mice with a RyR1 mutation. Nature communications 8: 14659 Doi 10.1038/ncomms14659

4 Lee JM, Rho SH, Shin DW, Cho C, Park WJ, Eom SH, Ma J, Kim DH (2004) Negatively charged amino acids within the intraluminal loop of ryanodine receptor are involved in the interaction with triadin. The Journal of biological chemistry 279: 6994-7000 Doi 10.1074/jbc.M312446200

5 Ngo VA, Perissinotti LL, Miranda W, Chen SRW, Noskov SY (2017) Mapping Ryanodine Binding Sites in the Pore Cavity of Ryanodine Receptors. Biophysical Journal 112: 1645-1653 Doi 10.1016/j.bpj.2017.03.014

6 Wei R, Wang X, Zhang Y, Mukherjee S, Zhang L, Chen Q, Huang X, Jing S, Liu C, Li Set al (2016) Structural insights into Ca(2+)-activated long-range allosteric channel gating of RyR1. Cell Research 26: 977-994 Doi 10.1038/cr.2016.99

7 Zhou H, Yamaguchi N, Xu L, Wang Y, Sewry C, Jungbluth H, Zorzato F, Bertini E, Muntoni F, Meissner Get al (2006) Characterization of recessive RYR1 mutations in core myopathies. Human molecular genetics 15: 2791-2803 Doi 10.1093/hmg/ddl221
